# Genome-Wide Methylation Profiling of Peripheral T–Cell Lymphomas Identifies TRIP13 as a Critical Driver of Tumor Proliferation and Survival

**DOI:** 10.3390/epigenomes8030032

**Published:** 2024-08-21

**Authors:** Pawel Nowialis, Julian Tobon, Katarina Lopusna, Jana Opavska, Arshee Badar, Duo Chen, Reem Abdelghany, Gene Pozas, Jacob Fingeret, Emma Noel, Alberto Riva, Hiroshi Fujiwara, Alexander Ishov, Rene Opavsky

**Affiliations:** 1 Department of Anatomy and Cell Biology, University of Florida College of Medicine, Gainesville, FL 32610, USA; 2 Department of Molecular Medicine, Long School of Medicine, University of Texas Health Science Center at San Antonio, San Antonio, TX 78229, USA; 3 Biomedical Research Center, Slovak Academy of Sciences, 84104 Bratislava, Slovakia; 4 UF College of Liberal Arts and Sciences, University of Florida, Gainesville, FL 32610, USA; 5 College of Agriculture and Life Sciences, University of Florida, Gainesville, FL 32610, USA; 6 ICBR Bioinformatics, Cancer and Genetics Research Complex, University of Florida, Gainesville, FL 32610, USA; 7 Department of Hematology, Clinical Immunology, and Infectious Diseases, Ehime University Graduate School of Medicine, Toon 791-0295, Japan

**Keywords:** cancer biology, DNA methylation, DNA methyltransferase, gene expression, lymphoma

## Abstract

Cytosine methylation contributes to the regulation of gene expression and normal hematopoiesis in mammals. It is catalyzed by the family of DNA methyltransferases that include DNMT1, DNMT3A, and DNMT3B. Peripheral T–cell lymphomas (PTCLs) represent aggressive mature T–cell malignancies exhibiting a broad spectrum of clinical features with poor prognosis and inadequately understood molecular pathobiology. To better understand the molecular landscape and identify candidate genes involved in disease maintenance, we profiled DNA methylation and gene expression of PTCLs. We found that the methylation patterns in PTCLs are deregulated and heterogeneous but share 767 hypo- and 567 hypermethylated differentially methylated regions (DMRs) along with 231 genes up- and 91 genes downregulated in all samples, suggesting a potential association with tumor development. We further identified 39 hypomethylated promoters associated with increased gene expression in the majority of PTCLs. This putative oncogenic signature included the *TRIP13* (thyroid hormone receptor interactor 13) gene whose genetic and pharmacologic inactivation inhibited the proliferation of T–cell lines by inducing G2-M arrest and apoptosis. Our data thus show that human PTCLs have a significant number of recurrent methylation alterations that may affect the expression of genes critical for proliferation whose targeting might be beneficial in anti-lymphoma treatments.

## 1. Introduction

Non-Hodgkin’s lymphomas (NHLs) are a heterogeneous group of lymphoid malignancies that arise from the transformation of B-, T-, and NK cells. Although the majority of NHLs are B-cell lymphomas (BCLs), ~15% of patients in Western countries suffer from more aggressive peripheral T–cell lymphomas (PTCLs) with poor survival rates [1,2,3]. PTCLs exhibit a broad spectrum of clinical, histological, and immunophenotypic features, which present significant therapeutic challenges and an overall unfavorable prognosis. Based on their manifestations—disseminated, nodal disease, extranodal, or cutaneous—and molecular features, at least 29 discrete types of PTCLs have been identified to date [4].

The three most common PTCL subtypes are anaplastic large cell lymphoma (ALCL), angioimmunoblastic T–cell lymphoma (AITL), and PTCL-not otherwise specified (PTCL-NOS).

Anaplastic large cell lymphoma is a group of aggressive lymphoproliferative disorders characterized by the expression of CD30 on lymphocytes. These disorders affect lymph nodes and various extranodal sites. ALCL is further classified based on the presence or absence of the anaplastic lymphoma kinase (ALK) protein into ALK-positive and ALK-negative subtypes, with significant differences in prognosis and molecular profiles between the two [5]. AITL arises from the follicular T helper cells and manifests by liver and spleen enlargement, lymphadenopathy, and weight loss [4,5].

Approximately 30% of PTCLs are “not otherwise specified” (PTCL-NOS), constituting the most common subtype. This group of high-grade, mature T–cell neoplasms is not classified by other WHO criteria due to our poor understanding of their molecular makeup [4]. Patients with PTCL-NOS are diagnosed with disease confined to the lymph nodes with frequent involvement of the liver, spleen, bone marrow, gastrointestinal tract, and skin. PTCL-NOS cells have an aberrant expression of T–cell markers (e.g., the lack of CD7 expression) with either CD4+/CD8- or CD4-/CD8+ phenotype and have a high proliferation rate [4,5]. Recently, two new subgroups of PTCL-NOS were identified as follows: (1) *TBX21* expressing (PTCL-TBX21; 49% of cases) and (2) *GATA3* expressing (PTCL-GATA3; 33% cases) [6].

Due to the poor understanding of the disease, peripheral T–cell lymphomas (PTCLs) are clinically treated with combination chemotherapy such as CHOP or CHOEP, but with limited success [4,5]. For example, PTCLs, have a low 32% 5-year survival rate [3].

Cytosine methylation in mammalian genomic DNA is an epigenetic mark that along with covalent histone modifications contributes to the suppression of gene promoter activity and repetitive elements resulting in transcriptional repression [7,8]. It can also lead to more efficient binding of transcription factors to their recognition sites as well as restricting the activation of alternative promoters within gene bodies, thereby promoting transcription [9,10]. The family of DNA methyltransferases (DNMT1, DNMT3A, DNMT3B, DNMT3L; DNMTs) is primarily responsible for catalyzing the addition of a methyl group to cytosines in CpG dinucleotides in DNA on a genome-wide level. DNMT1 is considered to be the main maintenance enzyme while DNMT3A and DNMT3B are primarily involved in de novo activities [11,12,13]. DNMT3L lacks catalytic activity but serves as a critical accessory protein for de novo methylation [14]. Several other proteins are important in the regulation of cytosine methylation. The TET enzymes are a family of methylcytosine dioxygenases and are critical for DNA demethylation. Another protein that can affect DNA methylation is the TCL1A protein, which inhibits DNMTs biochemically and affects global methylation in a mouse model of hematological malignancies [15].

Given the importance of methylation modifiers in the regulation of transcription, it is not surprising that many players are mutated in hematologic malignancies. Most point mutations in DNMT3A and TET2 are frequently found in hematologic malignancies including PTCL and result in loss of functions [16]. Consistently, loss of function mouse models demonstrated that DNMT3a, DNMT3b, and TET2 are tumor suppressors in a variety of hematologic malignancies including T- and B–cell lymphomas [17,18,19,20,21,22,23]. The molecular landscape of mouse lymphomas was characterized by global hypomethylation affecting all parts of the genomes including repeat elements, promoters, enhancers, gene bodies, and intergenic regions and contributing to de-repression. In contrast, locus-specific hypermethylation in promoters resulted in gene silencing of a large number of genes [17,18,19,20,21,22,23].

Comprehensive DNA methylation profiling can provide a wealth of information on molecular changes altered in tumors which, in turn, can be used for the development of diagnostic and prognostic markers, and lead to the identification of key drivers involved in the initiation and progression of PTCL. Given the value of such information, cancer-specific DNA methylation patterns of several human PTCL types have been partially characterized to date. In subtypes of human ALCL, DNA methylation signatures affect TCR and other genes involved in T–cell differentiation, as well as transcription factor induction and occupancy thereby contributing to ALCL oncogenic signaling [24]. In hepatosplenic TCL, several hyper- or hypomethylated loci were identified in gene regulatory elements including enhancers with eight-gene signatures of recurrent changes [25].

To better understand the recurrent molecular changes, and their possible contribution to disease maintenance, here we used high-resolution methylation and gene expression profiling of up to 10 PTCLs along with normal T–cells.

We found that whereas normal T–cell methylation patterns are quite similar across several samples, methylation patterns in PTCL are heterogeneous and characterized by both hypo- and hypermethylated regions relative to normal controls with the majority of changes not shared across tumors. In addition, we identified methylation (767 hypo- and 567 hypermethylated DMRs) and gene expression signatures (231 genes up- and 91 genes down) present in all tested tumors, suggesting a potential association with tumor development. We also found that putative tumor suppressors, such as *FOXP1*, *STK4*, and *ATM*, were frequently hypermethylated and silenced whereas oncogenes, such as *UHRF1*, *MPZL1*, *CDK14*, *RACGAP1*, and *RAB13*, were hypomethylated and overexpressed, suggesting that DNA methylation changes contribute to PTCL development. Among putative oncogenes, *TRIP13* (thyroid hormone receptor interactor 13) was also hypomethylated and overexpressed in 9/10 PTCLs on an average of ~10-fold. We show that both TRIP13 knockdown and its pharmacological inhibition by a small-molecule inhibitor DCZ0415 impaired cellular growth of T–cell lymphoma cell line T8ML-1 [26], as well as T–cell leukemia cell lines by inducing G2-M arrest and apoptosis suggesting the role of this gene in maintenance of T–cell malignancies.

Altogether, we show that human PTCL are characterized by a large number of recurrent methylation alterations affecting the expression of genes involved in tumorigenesis and functionally demonstrate that TRIP13 plays a role in PTCL lymphoma maintenance in vitro. Therefore, targeting TRIP13 might be beneficial in human PTCL treatment.

## 2. Results

### 2.1. DNA Methylome and Transcriptome of Normal Human CD4+ and CD8+ T–Cells

**Figure 1:**
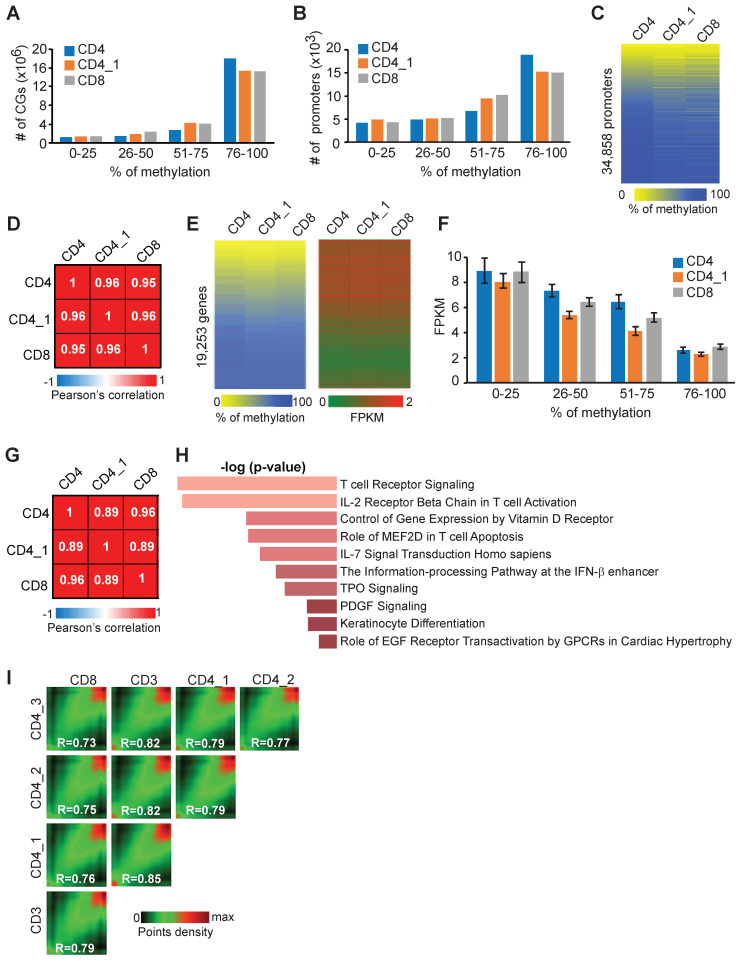
DNA methylome and transcriptome of normal human T–cells. (**A**) Breakdown of CpG methylation as determined by WGBS in two independent CD4+ (CD4, CD4_1) and one CD8+ (CD8) normal human T–cell samples. Individual CpGs were placed into four categories based on percent methylation (0–25%, 26–50%, 51–75%, and 76–100%). (**B**) Breakdown of promoter methylation for 34,858 genes in CD4+ and CD8+ normal human T–cell samples. Methylation percentages for all CpGs across the 2000 bp region (−1500 bp to +500 bp relative to TSS) were averaged to give a mean methylation value for each gene promoter. Promoters were placed into four categories based on percent methylation (0–25%, 26–50%, 51–75%, and 76–100%). (**C**) Methylation status of 34,858 promoters in CD4+ and CD8+ samples determined by WGBS. Mean promoter methylation was determined as described in (**B**). A scale is shown below the heat map, in which yellow and blue correspond to a lower and higher methylation status, respectively. (**D**) Pearson’s correlation coefficients (R values) derived from pairwise comparisons of 34,858 promoter methylation between CD4+, CD4_1, and CD8+ samples. Values > 0.9 indicate a strong positive correlation. (**E**) Heat map displaying the methylation status of 19,253 promoters in normal human CD4+ and CD8+ T–cells as determined by Whole Genome Bisulfite Sequencing (WGBS), analyzed using the same method described in (**B**), alongside corresponding gene expression levels (FPKM values obtained from RNA-seq analysis). A scale is shown below the heat map, in which yellow and blue correspond to a lower and higher methylation status, respectively. The corresponding expression is shown as a heat map with highly expressed genes denoted in red and lowly expressed genes denoted in green. (**F**) Analysis of promoter methylation and gene expression in normal human T–cells for 19,253 genes. Genes were divided into four groups based on the percentage of promoter methylation (0–25%, 26–50%, 51–75%, and 76–100%). Bars represent Mean +/−SEM FPKM. Pairwise comparisons were made for four methylation groups within each sample. All differences were statistically significant (*p* < 0.05, two-tailed Student’s *t*-test) except 0–25% vs. 26–50% and 26–50% vs. 51–75% in the CD4+ (blue) sample. (**G**) Pearson correlation coefficients (R values) derived from pairwise comparisons of gene expression values of 19,253 genes in normal CD4+ and CD8+ T–cells based on RNA-seq data. Values > 0.85 indicate a strong positive correlation. (**H**) Pathway enrichment analysis of 3001 highly expressed genes (FPKM ≥ 5) by *EnrichR.* The most significant pathways in “*Biocarta 2016*” are shown (*p* < 0.05). (**I**) Pairwise comparison of CpG methylation in normal CD4+ (*n* = 3), CD8+ (*n* = 1), and CD3+ (*n* = 1) T–cells. The density of points increases from green to red. R values represent Pearson’s correlation coefficients.

To identify molecular changes associated with PTCL development, we first sought to determine methylation and gene expression patterns in normal T–cells. To achieve that, we obtained CD4+ and CD8+ normal human T–cell samples isolated from peripheral blood (Precision Medicine) and subjected them to whole genome bisulfite sequencing (WGBS) and RNA-seq analysis. WGBS yielded more than 28 M reads for each sample (Figure S1). We next analyzed methylation data along with data obtained from purified CD4+ T–cells (sample CD4_1) for which both WGBS and RNA-seq were publicly available. The methylation analysis revealed that 21,936,712 individual CpG dinucleotides (CGs) were covered ≥5× in all three samples and were used further. A majority of CGs had a high level of methylation with more than 1.4 × 10^7^ CGs methylated to at least 75% in all three samples, whereas only 1 × 10^6^ CGs were methylated ≤ 25% (Figure 1A). Further analysis revealed that more than 15,000 out of 34,858 promoters (defined as −1500 bp to +500 bp relative to the transcription start site -TSS) were heavily methylated (at least 75%) and only ~4000 promoters were methylated at lower levels (less than 25%) in each sample (Figure 1B,C and Supplementary Materials S1). The positional variability in promoter methylation was low with very similar patterns across samples as determined by Pearson’s correlation with R values close to 1 in all pairwise comparisons (Figure 1D). We next utilized RNA-seq data to determine if methylation may impact gene expression. This analysis revealed that, in general, the degree of promoter methylation is inversely correlated with gene expression (Figure 1E,F and Supplementary Materials S2). Pairwise comparisons of gene expression and methylation revealed that FPKM values for genes with different percentages of promoter methylation were significantly different (*p* < 0.05, two-tailed Student’s *t*-test) in all comparisons except 0–25% vs. 26–50% and 26–50% vs. 51–75% in the first CD4+ T–cells sample (Figure 1F). For example, genes with promoters that were less than 25% methylated were expressed at higher levels than genes with highly methylated promoters (>75%; Figure 1F). Three analyzed T–cell samples were more variable in gene expression patterns because R values in pairwise comparisons were lower than those seen based on promoter methylation (Figure 1G). This result suggests that promoter methylome is more stable than transcriptome in normal T–cells across individuals and perhaps less prone to polymorphic changes in the human population.

Pathway enrichment analysis of 3001 highly expressed genes (FPKM ≥ 5) by *EnrichR* revealed, not surprisingly, significant enrichment in genes related to T–cell development. The most significant in *Biocarta 2016* were T–cell receptor, IL2-, IL7- signaling, and T–cell apoptosis (Figure 1H).

To further investigate methylation variability in human T–cells, we analyzed the similarity of methylation patterns among an additional three samples for which WGBS data are available. A pairwise comparison of the methylation status of 7,628,019 cytosines (covered 5×) that overlapped in all controls revealed that samples showed a high degree of similarity irrespective of their immunophenotypes (R = 0.73–0.85) for all pairwise comparisons (Figure 1I).

Our analysis thus demonstrates that a majority of CpG dinucleotides and promoters are mostly methylated in normal T–cells and that methylation polymorphism is limited resulting in relatively infrequent differences in promoter methylation between CD4+ and CD8+ T–cells. 

### 2.2. DNA Methylome of Peripheral T–Cell Lymphomas

**Figure 2:**
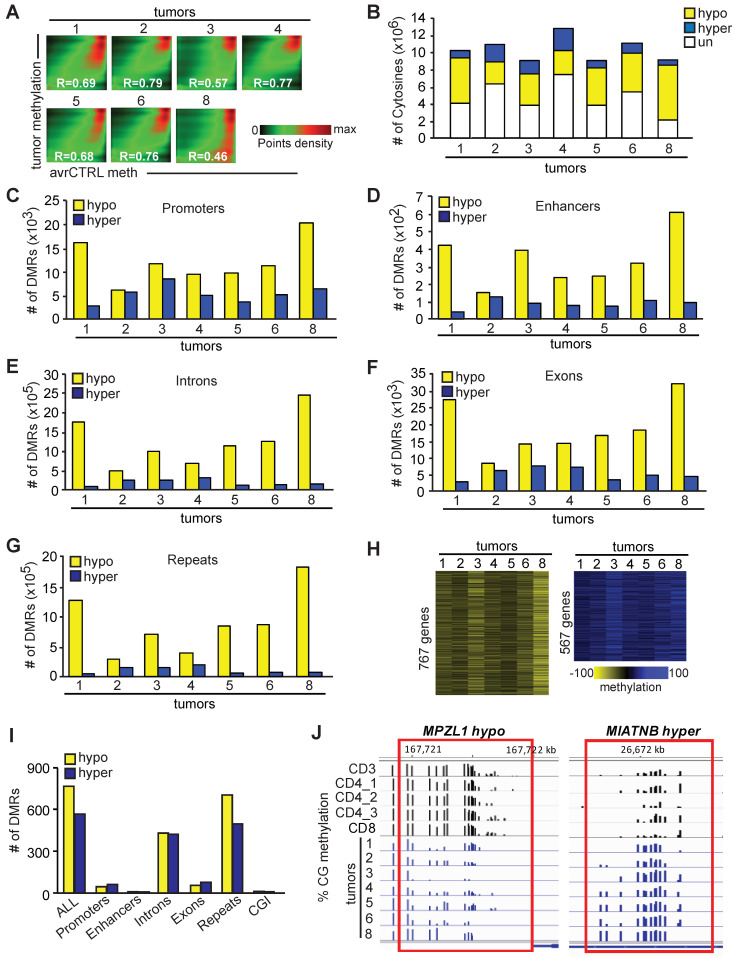
DNA methylome of human peripheral T–cell lymphomas. (**A**) Pairwise comparison of CpG methylation in human PTCLs against the average of five normal T–cell controls (CD3, CD4_1, CD4_2, CD4_3, CD8) averaged out by *Metilene*. The density of points increases from green to red. R values represent Pearson’s correlation coefficients. (**B**) The number of hypomethylated (yellow), hypermethylated (blue), and unchanged (white) CpGs in human PTCLs when compared to respective normal controls. (meth. diff. ≥ 10%). (**C**) The numbers of hypo- and hypermethylated DMRs in promoter regions of human PTCLs when compared to normal controls (DMRs; regions with methylation difference ≥ 10% in the same direction in ≥3 consecutive DMCs separated by <100 bp; p (MWU) < 0.05). The numbers of hypo- and hypermethylated DMRs in enhancers (**D**), introns (**E**)**,** exons (**F**), and repetitive elements (**G**) of human PTCLs when compared to normal controls. (**H**) Heat maps of the ‘Core PTCL Methylation Signature’ containing 767 hypo- and 567 hypermethylated DMRs present in all seven PTCLs tested. DMRs were analyzed as described in the (**C**) part of this figure. A scale of % methylation relative to the average of controls is shown below the heat map. Yellow and blue correspond to a lower and higher methylation status, respectively. (**I**) Distribution of DMRs in the ‘Core PTCL Methylation Signature’ across indicated genomic elements. (**J**) Percentage of methylation at individual CpGs in DMRs at *MPZL1* locus (left) and *MIATNB* (right) at indicated genomic coordinates consistently hypo- and hypermethylated in PTCLs, respectively, as visualized by IGB software 10.0.1.

To analyze DNA methylation in human PTCL, we first obtained a set of seven primary human lymphoma samples from both the Cooperative Human Tissue Network (CHTN) and commercial sources. This resulted in a collection of four ALCLs (T1–T4), one AITL sample (T5), and two PTCL-NOSs (T6, T8) (Figure S2). After DNA isolation, we subjected the obtained samples to global methylation profiling using WGBS. Analysis of the data revealed that we were able to obtain sequencing depths ranging from 16.5 to 24 M CGs that were covered by at least five sequence reads in each sample (Figure S3). For the analysis of tumor-specific methylation, we used a control sample consisting of methylation data obtained from five normal human T–cells—CD3, CD4_1, CD4_2, CD4_3 and CD8—averaged out by *Metilene.* A pairwise comparison of the methylation status of 7,628,019 cytosines (covered 5×, averaged out by *Metilene*) that overlapped in all samples revealed that tumors were severely hypomethylated relative to the controls (Figure 2A, Figures S1 and S3). This was further confirmed by the analysis of differentially methylated cytosines (DMCs), which were those that had at least 10% methylation change relative to controls (*p* < 0.05 (MWU)). Our pairwise analysis of CGs covered in all samples revealed that most of the DMCs were hypomethylated relative to normal T–cells in all tumors (Figure 2B). Hypomethylation was most pronounced in T1 and T8 lymphomas and the least in T2 and T4, which had almost equal numbers of hypo- and hypermethylated DMCs (Figure 2B). More stringent analysis of methylation changes using differentially methylated regions (DMRs; defined as ≥10% methylation change in the same direction in three consecutive cytosines in ≤100 bp, *p* < 0.05 (MWU)) confirmed the large-scale deregulation of methylation in all tumors. Notably, thousands of hyper- and especially hypomethylated DMRs were observed in all tumors in various genomic elements including promoters, enhancers, introns, exons, and repeats (Figure 2C–G). Like in mouse hematologic malignancies [17,18,19], the presence of hypo- rather than hypermethylated DMRs in promoters was a more frequent event in all tumors ranging from ~6000 (T2) to ~20,000 (T8) (Figure 2C). However, promoter hypermethylation was seen in all tumors and ranged from ~2000 (T1) to 8000 (T3). In some tumors—T2 and T3—promoter hypo- and hypermethylation were almost equal in frequency (Figure 2C). Patterns of hypo- and hypermethylation changes did not appear to depend on tumor type since the most hypomethylated tumors—T1 and T8—belonged to different PTCL subtypes (ALCL and PTCL-NOS, respectively).

While tumors varied in the frequency of hypo- and hypermethylated DMRs in genomic elements, the trend remained similar within individual tumors. For example, the most hypomethylated tumors were T1 and T8, and hypomethylation was manifested across all genomic elements. Similarly, the ratio between hypo- and hypermethylated DMRs was the smallest in T2, and that trend was seen across all genomic elements (Figure 2C–G).

Not surprisingly, the biggest ratio favoring hypo- over hypermethylated DMRs was observed in repetitive elements that are known to be prone to loss of methylation in tumors (Figure 2G). Interestingly, as many as 767 hypo- and 567 hypermethylated DMRs were recurrently observed in all tumors tested (Figure 2H,I, Figure S4 and Supplementary Materials S3). We consider these high-frequency DMRs as representing the ‘Core PTCL Methylation Signature’. These DMRs were distributed in various genomic elements but especially in repeats and introns (Figure 2I and Figure S5). For example, *MPZL1* and *UCK2* loci uniformly hypermethylated in normal T–cells were hypomethylated in lymphomas with high frequency (Figure 2J and Figure S6). Similarly, *MIATNB* and *TXK* loci hypomethylated in normal T–cells gained methylation in all tested lymphomas. (Figure 2J and Figure S6). Among other notable genes with promoter hypermethylation were *LCK*, *CD101*, *PGGHG*, *RASSF3*, and *SLC39A2* genes, and with promoter hypomethylation *PTAFR*, *MPZL1*, *PRDM11*, *CTNND1*, *FLI1*, *GIT2*, *EML5*, *RBFOX1*, *KSR1*, and others (Supplementary Materials S3).

Further dissection of this signature may reveal methylation markers of PTCL associated with the disease initiation and progression, as well as genes driving the disease development.

### 2.3. Analysis of Gene Expression in ALCL and PTCL-NOS

**Figure 3:**
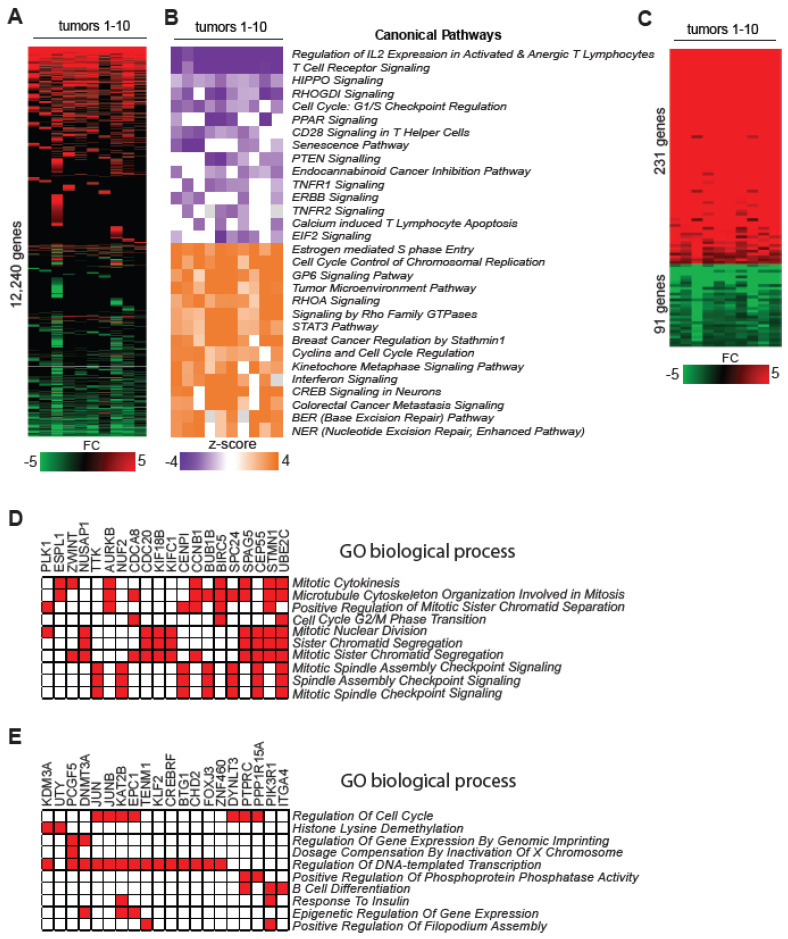
Gene expression signatures and deregulated pathways in PTCLs. (**A**) Heat map showing fold change expression values of a subset of differentially expressed genes (DEGs; FC ≥ 1.5, adjusted *p* < 0.05 by DESeq2) in human T–cell lymphomas (*n* = 10) relative to control T–cells (*n* = 4) as analyzed by RNA-seq. A scale is shown below the heat map, in which green and red correspond to a decrease and increase in expression, respectively. (**B**) Results of ingenuity pathway analysis (IPA) performed on all DEGs in individual tumors. The top 15 up- and downregulated canonical pathways that were most consistently deregulated in analyzed human lymphomas are shown. (**C**) ‘Core PTCL Gene Expression Signature’ consisting of 231 up- and 91 downregulated genes relative to the average of normal T–cells (*n* = 4) in all 10 tested lymphomas as determined by RNA-seq data analysis. (**D**) Pathway enrichment analysis of upregulated genes in the ‘Core PTCL Gene Expression Signature’ in the category GO Biological Process by EnrichR (*p* < 0.05). (**E**) Pathway enrichment analysis of downregulated genes in the ‘Core PTCL Gene Expression Signature’ in the category GO Biological Process by EnrichR (*p* < 0.05).

To determine the extent to which methylation changes may contribute to deregulated transcriptome in PTCL, we next performed global gene expression profiling of PTCL samples using RNA-seq analysis. In addition to PTCL samples used for methylation analysis, we also included RNA isolated from three additional PTCL-NOS samples (T7, T9, T10) along with RNA from normal T–cells used for methylation analysis (Figure 1, Figures S1 and S2). These samples were subjected to RNA-seq analysis and the data were analyzed using *SeqMonk* software 1.47.2 and the DeSeq2 method to calculate differential expression. Our analysis revealed large-scale deregulation in transcriptomes of PTCL samples relative to normal T–cells manifested by findings that 12,240 genes were deregulated in at least one tumor relative to the averaged-out values of independent normal T–cell samples (Figure 3A and Figure S2 and Supplementary Materials S4).

Ingenuity pathways analysis (IPA) of differentially expressed genes [FC ≥ 1.5, *p* < 0.05 by DESeq2) in human T–cell lymphomas (n = 10) relative to control T–cells (n = 4)] revealed that all PTCLs had inhibited pathways related to Regulation of IL-2 Expression in Activated and Anergic T Lymphocytes, T Cell Receptor Signaling, and HIPPO signaling (Figure 3B). Additional frequently suppressed pathways included RHOGDI Signaling, Cell Cycle G1/S Checkpoint Regulation, tumor-suppressive PTEN Signaling, and others (Figure 3B). In contrast, pathways frequently activated in all tested samples included Estrogen-mediated S-phase Entry, Cell Cycle Control of Chromosomal Replication, Signaling by Rho Family GTPases, and the STAT3 pathway. Additionally, there was a high frequency of activation in Interferon and RHOA Signaling pathways, along with other cancer-related pathways (Figure 3B).

The expression of 231 genes was increased and the expression of 91 genes was decreased at least 2-fold in all 10 PTCLs. We term these molecular changes as ‘Core Gene Expression Signature’ (Figure 3C and Supplementary Materials S5). The top five genes overexpressed in all PTCL samples encode the following: phospholipase PLA2G2D, organic anion transporting polypeptide SLCO2B1, metalloprotease ADAMDEC1, complement protein C1QB, and receptor activity modifying protein RAMP3, with the average increase in expression over 500-fold (Supplementary Figure S7 and Supplementary Materials S5). None of these genes seem to have an established role in T–cell transformation and their deregulation can be a consequence of changes in signaling and epigenetic alterations in PTCL. GO enrichment analysis identified pathways linked to cell cycle regulation and in particular to Cell Cycle G2/M Phase Transition, Mitotic Nuclear Division, and Sister Chromatid Segregation (Figure 3D). Analysis of genes not directly linked to the cycle revealed pathways related to Complement Activation, Angiogenesis, Blood Coagulation, and Notch Pathway Signaling (Figure S8). ChIP enrichment analysis revealed the possible involvement of FOXM1, E2F1, E2F4, SOX2, KLF4, and MYBL2 transcription factors in the pathogenesis of PTCL because they may play a role in the deregulation of the ‘Core PTCL Expression Signature’ (Figure S9).

In all 10 PTCL samples, 91 genes exhibited a consistent decrease in expression, with at least a 2-fold reduction (Figure 3C). Notably, the most frequently downregulated genes included *FGFBP2* (also known as *KSP37*), teneurin *TENM1*, and a group of Y-linked genes such as *USP9Y*, *TTTY15*, and *UTY* (Figure 3C and Figure S7). Their role in lymphomagenesis remains unclear.

The GO enrichment analysis identified pathways linked to the Cell cycle, Histone demethylation, epigenetic dysregulation, and B cell differentiation likely occurring with the contribution of transcription factors FOXO1, FOXOM1, MYB, and others (Figure 3E, Figures S10 and S11).

Upregulation of *TBX21* or *GATA3* and their target genes (*EOMES*, *CXCR3*, *IL2RB*, *CCL3*, *IFNγ*, and *CCR4*, *IL18RA*, *CXCR7*, *IK*, respectively) was previously shown to distinguish two subclasses of PTCL-NOS with distinct clinical outcomes [6,27]. To determine if any of the five samples included in our analysis belong to a specific PTCL-NOS subtype, we analyzed RNA-seq expression patterns further. However, we did not see upregulation of *TBX21* or *GATA3* when compared to normal controls in any of the five cases of PTCL-NOS, perhaps because our analysis was limited by the small sample size (Figure S12).

### 2.4. DNA Methylation Modifiers Are Deregulated in Human PTCL

**Figure 4:**
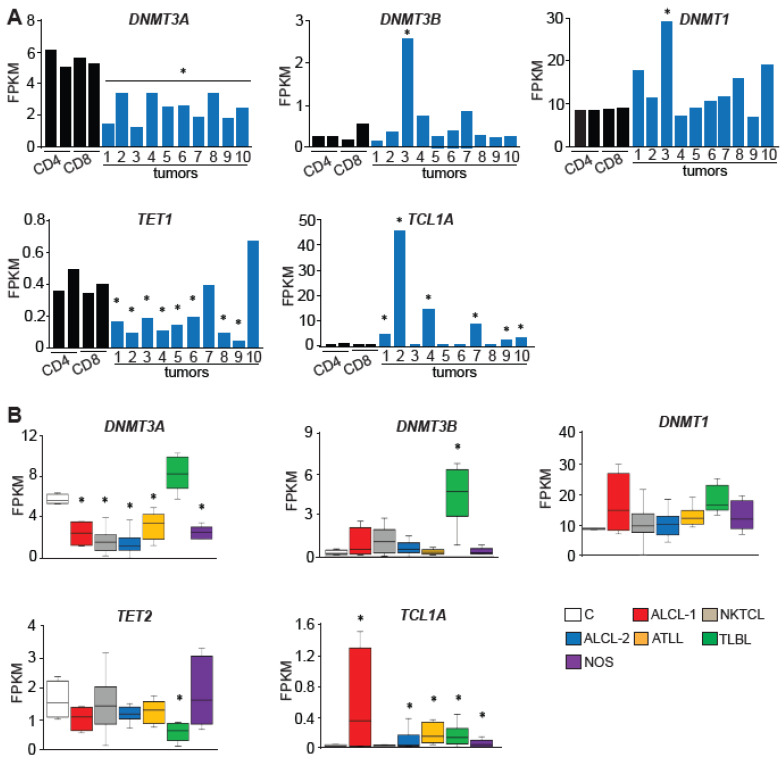
Expression of DNA methylation modifiers is deregulated in human PTCLs. (**A**) Expression of *DNMT3A*, *DNMT3B*, *DNMT1*, *TET1*, and *TCL1A* in human PTCLs and control T–cells as analyzed by RNA-seq. Statistical analysis was performed by comparison of individual tumors to average values obtained from control T–cells (*n* = 4). The statistically significant differences (*p* < 0.05 by DESeq2) are indicated by *. (**B**) *DNMT3A*, *DNMT3B*, *DNMT1*, *TET2*, and *TCL1A* expression as determined by RNA-seq analysis of normal human T–cells (C, *n* = 4), two sets of human anaplastic large cell lymphomas (ALCL-1, n = 5 tumors T1–T5 profiled in this study; ALCL-2, *n* = 21 publicly available data), NKTCL T–cell lymphoma lines (NKTCL, *n* = 15), adult T–cell leukemia/lymphomas (ATLL, *n* = 8), T-lymphoblastic lymphomas (TLBL, *n* = 8) and PTCL-NOS (*n* = 5, tumors T6-T10 profiled in this study). The horizontal line represents the median, bounds of the box-like range of variation, and whiskers min and max values. * *p* < 0.05 (by DESeq2).

To determine if levels of DNA methylation modifiers are deregulated in human PTCL, we further analyzed RNA-seq data and found that *DNMT3A* was significantly reduced in all tumors when compared to either CD4+ or CD8+ normal T–cells from peripheral blood (Figure 4A). In contrast, *DNMT1* and *DNMT3B* were unchanged with only one ALCL tumor, T3, showing a significant increase in transcript levels (Figure 4A). Analysis of DNA demethylases showed that *TET1* transcripts were low in all samples (FPKM < 1) but still significantly downregulated in the majority of tested tumors, whereas levels of *TET2* and *TET3* were unchanged relative to normal thymocytes (Figure 4A and Figure S13). Another protein that can affect DNA methylation is the TCL1A protein, which was shown to inhibit DNA methyltransferases biochemically and affect global methylation in a mouse model of hematological malignancies [15]. We, therefore, analyzed its expression and found 6 out of 10 analyzed tumors had significantly increased levels of *TCL1A* RNA relative to normal T–cells (Figure 4A). In contrast, the expression of another member of the TCL family—*TCL1B*—that does not inhibit DNMTs [15] was unchanged in PTCL (Figure S13).

To determine whether deregulated expression of methylation modifiers is more broadly observed in PTCL subtypes, we next utilized publicly available data generated by RNA-seq analysis of 15 primary natural killer/T–cell lymphomas (NKTCLs), 21 ALCLs (ALCL-2), 8 adult T–cell leukemia/lymphomas (ATLLs), 8 T-lymphoblastic lymphomas (TLBLs), and our 5 NOS and 4 ALCLs (ALCL-1). While the expression of *DNMT1* was mostly unchanged, *DNMT3A* levels were significantly reduced across all PTCL subtypes except TLBL (Figure 4B). Like *DNMT1*, *DNMT3B* expression was unchanged across most PTCLs except for a significant increase in TLBL (Figure 4B). The levels of *TET1* RNA showed a tendency for downregulation in a majority of analyzed PTCLs, although it did not reach statistical significance, while *TET3* was mostly unchanged irrespective of tumor type (Figure S14).

*TCL1A* was upregulated in a majority of PTCLs, whereas *TCL1B* was not significantly increased in any of the tested tumor subtypes (Figure 4B and Figure S14).

Altogether, this analysis revealed that several DNA methylation modifiers are deregulated in PTCL, with the downregulation of *DNMT3A* and *TET1* and the upregulation of *TCL1A* in most tested samples. These molecular changes and genetic alterations found in these modifiers in a subset of PTCL as reported by others [28,29,30] are the most likely reasons for the large-scale deregulation of PTCL methylomes that we observed.

### 2.5. Deregulated Promoter Methylation Is Associated with Changes in Gene Expression

To determine the extent to which methylation changes in PTCL may affect transcription, we further compared the data sets generated by WGBS and RNA-seq. First, we found that methylation changes also affected the expression of various repetitive elements, including DNA transposons, LTR and Non-LTR retrotransposons, and satellite repeats (Figure S15). In a subset of repeat elements, such as *UCON80*, *LTR39*, and *LTR180*, hypomethylation mostly correlated with an increased expression. In contrast, hypomethylation of *UCON34* correlated with decreased, rather than increased, expression (Figure S15). However, we didn’t detect any consistent pattern of methylation changes, gene expression, or their correlation in examined tumors, suggesting that repeats may not serve as good markers of disease development or progression.

**Figure 5:**
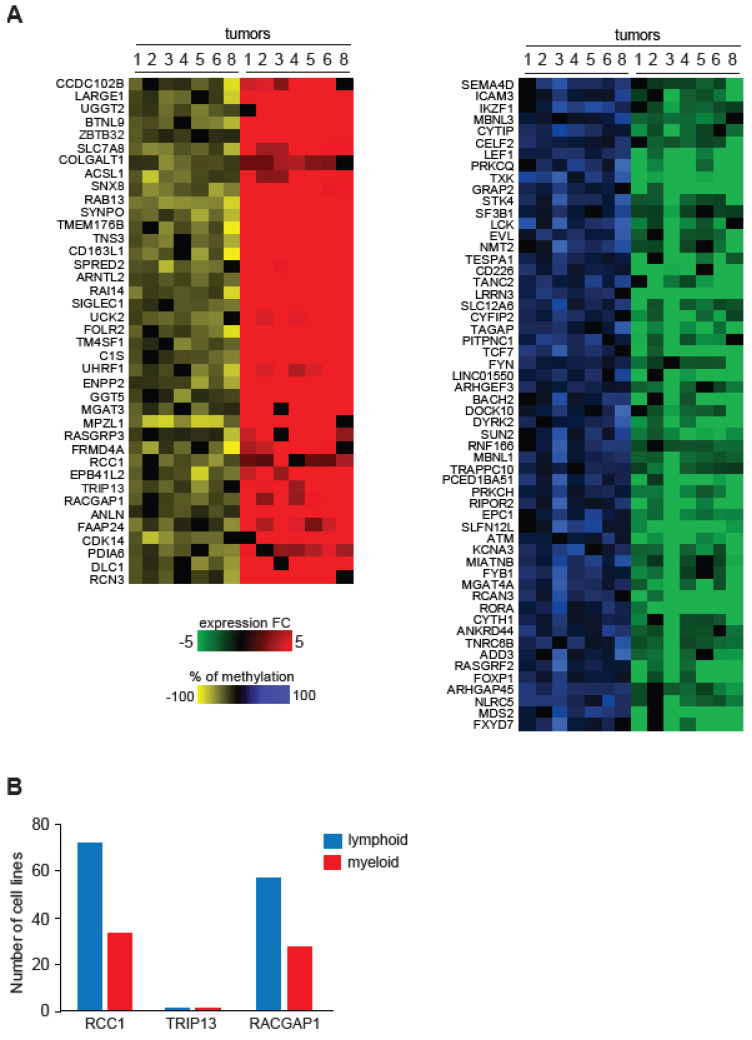
Promoter methylation is associated with changed gene expression for a subset of genes. (**A**) Left panel. Heat map displaying 39 genes with hypomethylated promoters (presence of one or more hypomethylated DMRs in −1500 bp to +500 bp) at a frequency at least six out of seven PTCLs and whose expression is increased relative to normal T–cell controls (≥2-fold change, *p* < 0.05) as determined by RNA-seq analysis. Numbers indicate individual tumors, the extent of hypomethylation is shown in yellow and hypermethylation in blue, while overexpression is shown in red and under expression in green. Right panel. Heat map displaying 56 genes with hypermethylated promoters (presence of one or more hypermethylated DMRs in −1500 bp to +500 bp) at a frequency of at least six out of seven PTCLs and that were also under-expressed relative to normal T- cell controls (≥2-fold change and a *p* value < 0.05) as determined by RNA-seq analysis. (**B**) The number of lymphoid (blue) and myeloid (red) cell lines for which the knockout of *RCC1*, *TRIP13*, or *RACGAP1* genes was lethal. The numbers were determined using DepMap portal’s (Broad Institute) perturbation effects analysis and the gene effect was considered lethal if the expression log2(TPM+1) was ≤−1.

Next, we examined gene expression changes that correlated with methylation changes in gene promoters at high frequency and found that 2810 genes contained hypomethylated DMRs in five out of seven tumors (Supplementary Materials S6). Out of those, 153 genes had increased expression in five out of seven tumors (Figure S16). EnrichR pathway enrichment analysis coupled with Wiki Pathway data sets revealed pathways regulating multiple cellular processes that are commonly upregulated in cancers, such as VEGFA-VEGFR2 Signaling, PI3K-Akt signaling, and EGF/EGFR signaling (Figure S17). Interestingly, several pathways related to Hippo signaling such as Hippo-Merlin Signaling Dysregulation, Overview of leukocyte-intrinsic Hippo pathway, Pathways Regulating Hippo, and Hippo-Yap signaling pathway raising the possibility that the deregulation of this pathway in T–cells could play a functional role in transformation. Promoters of 1281 genes contained hypomethylated DMRs in at least six out of seven tumors. Out of these, 39 genes were associated with increased expression (≥1.5) in PTCL, suggesting that a loss of methylation may have contributed to their deregulation (Figure 5A).

Several genes from this group, such as *UHRF1*, *MPZL1*, *CDK14*, *RACGAP1*, *RAB13*, and others, have oncogenic functions in various solid tumors, leukemias, and lymphomas either predicting poor prognosis, involved in tumor migration, metastasis, or maintenance [31,32,33,34,35].

We identified 1220 hypermethylated genes with the frequency of five out of seven tumors out of which 74 are also downregulated in five out of seven tumors (Supplementary Materials S6 and Figure S18). Decreased expression of these genes was associated with the deregulation of various pathways including the Wnt Signaling Pathway and Pluripotency and Pathways Regulating Hippo Signaling. (Figure S19). Promoters of 578 genes showed hypermethylated DMRs with a high frequency of six out of seven PTCL. Out of these, the expression of 56 genes decreased by at least 1.5-fold. (Figure 5A). Among these were genes with putative tumor suppressor function in different cancer types including *FOXP1*, *STK4*, *ATM*, and others [36,37,38].

Interestingly, several genes with potential oncogenic functions, such as *SF3B1*, *FYN*, and *LCK*, are also frequently downregulated but the physiological relevance of these changes for PTCL development remains unclear.

Our data show a relatively high number of recurrent methylation events observed in PTCL correlate with changes in gene transcription. Given that changes in expression affect many genes involved in various aspects of tumor biology, DNA methylation changes likely contribute to PTCL development in a causative way.

### 2.6. Loss of DNA Methylation Correlates with Upregulation of Genes Critical for Cancer Cell Proliferation

**Figure 6:**
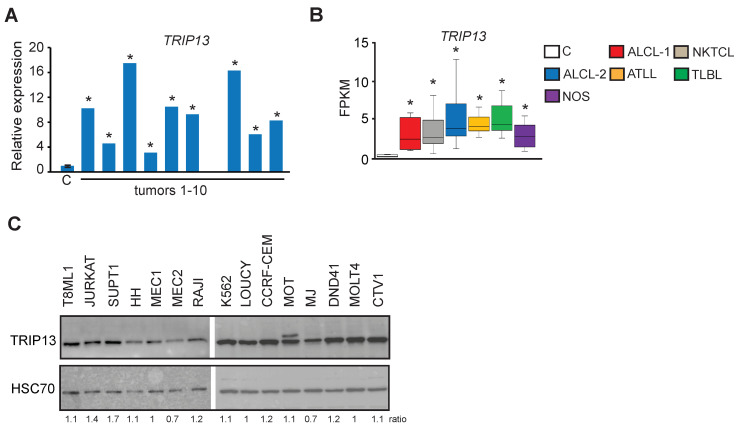
*TRIP13* is frequently upregulated in primary human PTCL. (**A**) Relative *TRIP13* expression as determined by RNA-seq. Data are presented as fold differences in FPKM values relative to the mean of normal T–cells (C; n = 4) obtained by RNA-seq analysis. Statistically significant differences are indicated by *. (**B**) *TRIP13* expression as determined by RNA-seq analysis of normal human T–cells (C, *n* = 4), two sets of human anaplastic large cell lymphomas (ALCL-1, *n* = 5 tumors T1-T5 profiled in this study; ALCL-2, *n* = 21 publicly available data), natural killer T–cell lymphomas (NKTCL, *n* = 15), adult T–cell leukemia/lymphomas (ATLL, *n* = 8), T-lymphoblastic lymphomas (TLBL, *n* = 8) and PTCL-NOS (*n* = 5, tumors T6-T10 profiled in this study). The horizontal line represents the median, bounds of the box-like range of variation, and whiskers min and max values. Pairwise comparisons between each tumor group and controls are statistically significant (*p* < 0.05 by DESeq2) indicated by *. (**C**) Immunoblot analysis of TRIP13 protein levels in human lymphomas and leukemia cell lines. T8ML-1 (PTCL-NOS); JURKAT (acute T–cell leukemia); SUP-T1 (T–cell lymphoblastic lymphoma); HH (cutaneous T–cell leukemia/lymphoma), MEC1, MEC2 (chronic B-cell leukemia); RAJI (Burkitt’s lymphoma); K-562 (chronic myelogenous leukemia); LOUCY, CCRF-CEM (acute T–cell lymphoblastic leukemia); Mo T (hairy T–cell leukemia); MJ (cutaneous T–cell lymphoma); DND41 (acute T–cell lymphoblastic leukemia); MOLT4 (T lymphoblast cell); CTV-1 (T-ALL). HSC70 served as a loading control.

To further explore whether promoter hypomethylation may affect aspects of T–cell lymphomagenesis, we next focused on the analysis of 39 genes hypomethylated and overexpressed with a high frequency in PTCL (Figure 5A). To determine if any of these genes may play a role in PTCL maintenance, we utilized data from CRISPR knockout screens in the DepMap database (Broad Institute, (39, 40)) and investigated whether any of the genes affected the growth of hematologic cell lines. This search revealed that knockouts of most genes did not significantly impact the proliferation of hematological cell lines, as indicated by the gene effect scores ranging from −1 to +1 (Figure 5B and Figure S20). In contrast, the knockout of *RACGAP1* and *RCC1* genes was lethal to the majority of cell lines, and they were therefore classified as Common essential required for proliferation of cells in general. Interestingly, TRIP13 (thyroid hormone receptor interactor 13) characterized by DepMap as Strongly selective was critical for the maintenance of several cancer cell lines including B–cell lymphoma OCILY19 and human eosinophilic leukemia EOL1. Because *TRIP13* is not a Common essential and therefore its targeting may be less toxic, we therefore focused on the analysis of this gene and sought to further explore its role in lymphomagenesis by determining its expression in primary TCL. Our analysis revealed that the gene was overexpressed in 9/10 PTCLs on average by ~10-fold relative to controls (Figure 6A). Analysis of publicly available RNA-seq data revealed overexpression of *TRIP13* in ALCL, ATLL, NKTCL, TLBL, and NOS (Figure 6B).

We next performed an immunoblot analysis of various cell lines to determine if the TRIP13 protein is present in hematologic cell lines. This analysis revealed that TRIP13 was expressed in T–cell lymphoma (T8ML-1, MJ, HH), T–cell leukemia (JURKAT, MOLT4, MOT, SUPT1, LOUCY, CCRF-CEM, DND-41), B–cell lymphoma (RAJI), B-cell leukemia (MEC-1, MEC-2), and myeloid leukemia (K562, CTV-1) cell lines (Figure 6C, Figures S21 and S22). Altogether, our data show that TRIP13 is overexpressed in primary TCL and expressed in various hematologic cell lines and may be involved in their maintenance.

### 2.7. TRIP13 Downregulation Inhibits the Proliferation of Malignant T–Cells

**Figure 7:**
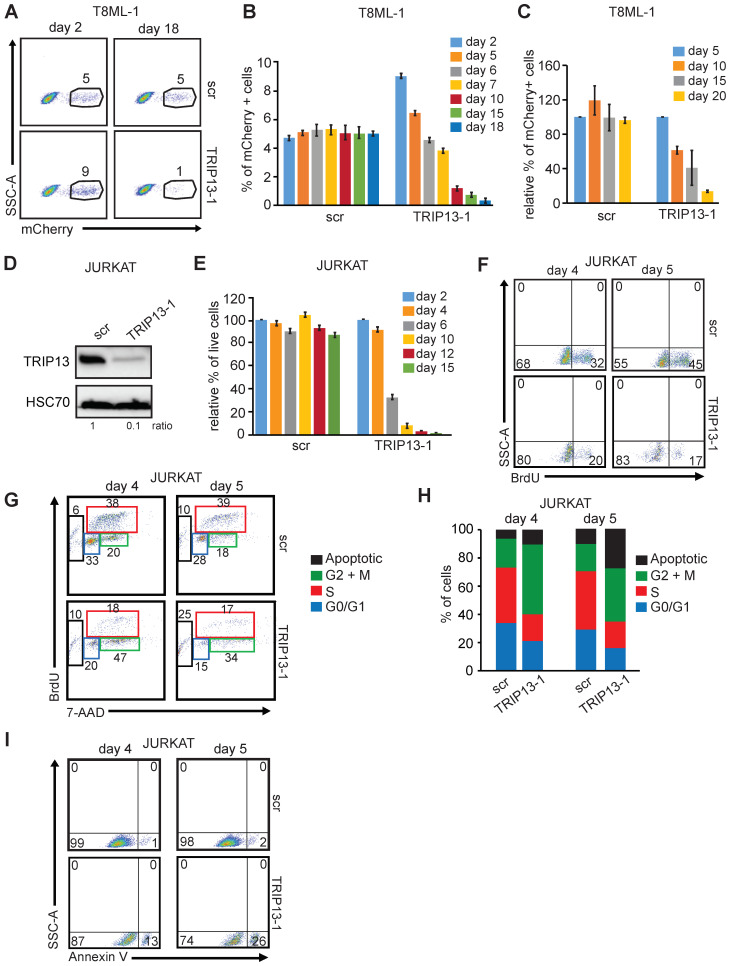
*TRIP13* downregulation results in impaired cellular proliferation of malignant T–cells. (**A**) Representative examples of FACS diagrams indicating mCherry expression measured in unselected T8ML-1 cell line transduced with lentiviruses expressing shRNA against either scrambled (scr) or *TRIP13* (shRNA-1). Data obtained at days 2 and 18 upon continuous culturing in vitro are shown. The percentage of mCherry positive cells is shown above the gated population. (**B**) Percentage of mCherry-positive cells expressing shRNA-1 against either scrambled or *TRIP13* upon continuous culturing in vitro at indicated times as measured by FACS. Data are presented as mean ± SEM (from three independent experiments) relative to scrambled, *p* < 0.05. *p* values were calculated by a two-tailed Student’s *t*-test. (**C**) Relative mCherry expression in T8ML-1 cells transduced with lentivirus encoding shRNA-1 against *TRIP13* or scrambled, measured by FACS 48 h after transduction (day 2) and 20 days later upon continuous culturing in vitro. The transduction efficiency, as measured by mCherry expression at day 2 was set to 100%, for both cells transduced with scrambled and *TRIP13* shRNAs. The values obtained for the percentage of mCherry-positive cells at each time point were plotted relative to the percentage at day 2. Data are presented as mean ±SEM (from three independent experiments) relative to scrambled, *p* < 0.05. *p* values were calculated by a two-tailed Student’s *t*-test. (**D**) Immunoblot analysis of TRIP13 protein levels in JURKAT cells transduced with lentiviruses expressing either scrambled or *TRIP13* shRNAs. Cells were harvested and analyzed 72 h after transduction. HSC70 served as a loading control. (**E**) Relative percentage of live unselected JURKAT cells transduced with the indicated lentiviruses at 99% efficiency as measured by FACS analysis of mCherry expression coexpressed from the lentiviral constructs. Viability was determined as the percentage of cells in the “live gate” in forward scatter FACS diagrams at indicated times. The values obtained for the percentage of mCherry-positive cells at each time point were plotted relative to the percentage at day 2. (**F**) Representative examples of FACS diagrams obtained from BrdU incorporation assays of JURKAT cells transduced with lentivirus expressing shRNA against either scrambled or *TRIP13* as determined at 4 and 5 after transduction. Cells were exposed to BrdU for 60 min, harvested and FACS analysis was used to evaluate the percentage of cells that incorporated BrdU. The percentage of positive cells in the FACS profile is shown within each plot. A representative example of two independent experiments is shown. (**G**) Representative examples of FACS diagrams obtained from cell cycle analysis of JURKAT cells transduced with lentivirus expressing shRNA against either scrambled or *TRIP13* as determined at 4 and 5 days after transduction. A combination of BrdU incorporation assay and staining with 7-ADD followed by FACS was used. (**H**) Percentage of cells in various phases of the cell cycle in JURKAT cells transduced with lentiviruses expressing shRNA against either scrambled or *TRIP13*, determined 4 and 5 days by BrdU incorporation assay coupled with 7-AAD staining and FACS-based analysis. (**I**) Representative examples of FACS diagrams obtained from Annexin V assays of JURKAT cells transduced with lentivirus expressing shRNA against either scrambled or *TRIP13* as determined at 4 and 5 days after transduction. The percentage of positive cells in the FACS profile is shown within each plot and indicates ongoing apoptosis.

The AAA ATPase TRIP13 (thyroid hormone receptor interactor 13) is known to participate in various regulatory steps related to the cell cycle, such as the mitotic spindle assembly checkpoint and meiotic recombination, as well as the DNA repair by immediate-early DNA damage sensing and ATM signaling activation [39]. To determine if TRIP13 is required for the proliferation of PTCL, we transduced a PTCL-NOS cell line, T8ML-1, with lentiviruses expressing shRNAs either targeting *scrambled* or *TRIP13* and coexpressing mCherry. Cells were cultured over 20 days and the percentage of mCherry-positive cells was measured at different time points by FACS. The percentage of cells expressing *scrambled* shRNA remained relatively stable, suggesting that the expression of *scrambled* shRNA or mCherry did not affect the proliferation of cells (Figure 7A,B). In contrast, the percentage of *TRIP13* shRNA-1 expressing cells was gradually reduced over time, suggesting that the proliferation of T8ML-1 cells is impaired by *TRIP13* downregulation. (Figure 7A–C). The proliferative defect was reproducible and was also seen using independent *TRIP13* shRNA-2 (Figure S23). We next asked whether *TRIP13* knockdown can affect the proliferation of T–cell leukemia JURKAT cells. These cells could be routinely transduced to more than 99% efficiency, thus allowing for direct cellular and molecular analysis (Figure S24). As expected, both *TRIP13*-specific shRNAs efficiently decreased protein and RNA levels of TRIP13 (Figure 7D, Figures S25 and S26). Similar to T8ML-1 cells, *TRIP13* knockdown resulted in impaired proliferation and reduced viability of JURKAT cells as determined by the decreased percentage of cells in “*live gate*” in FACS analysis and cell counts upon culturing over time (Figure 7E). *TRIP13* reduction resulted in decreased BrdU incorporation, G2-M arrest, increased Annexin V expression, and apoptosis (Figure 7F–I). In contrast to downregulation, lentivirally mediated overexpression of C-terminally FLAG-tagged TRIP13 CDS from lentiviral backbone coexpressing fluorescent protein in T8ML-1 did not affect the cell growth of T8ML-1 or JURKAT cells because the percentage of EGFP+ cells remained relatively stable over time in both TRIP13 and control cells. Altogether, these data suggest that the downregulation of *TRIP13* has antiproliferative effects by inducing G2-M arrest accompanied by apoptosis.

### 2.8. Treatment of T8ML-1 Cells with TRIP13 Inhibitor DCZ0415 Impairs Proliferation and Induces Cell Death

**Figure 8:**
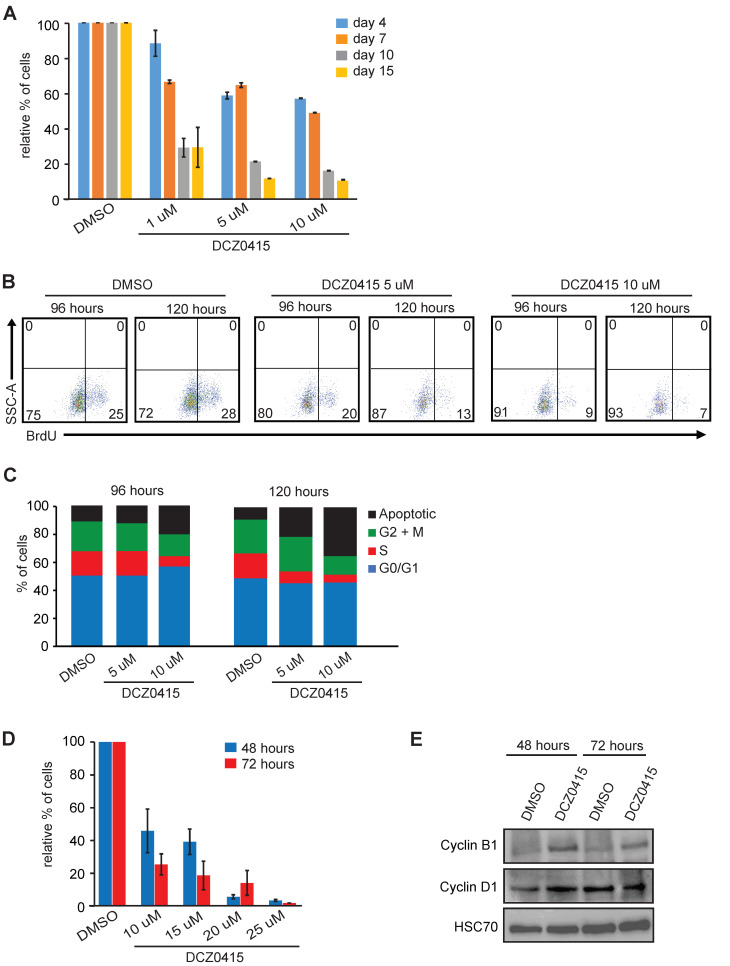
The treatment of T8ML-1 cells with DCZ0415 inhibits cellular growth by inducing G2-M arrest and cell death. (**A**) T8ML-1 cells were treated with either DMSO (vehicle) or DCZ0415 at indicated concentrations for 15 days and counted using Trypan Blue exclusion dye. Bar graphs represent the relative percent count of live cells after 4, 7, 10, and 15 days of continuous drug treatment. Each data point is the average of three measurements taken in parallel and bar graphs represent ±SEM. (**B**) Representative FACS diagrams of the BrdU incorporation assay of T8ML-1 cells treated with either DMSO, 5 or 10 μM of DCZ0415 after 96 and 120 h. Numbers in corners of FACS diagrams represent percentages obtained from quadrant statistics using FlowJo X software. (**C**) Percentage of cells in various phases of the cell cycle in T8ML-1 cells treated with either DMSO, 5 or 10 μM of DCZ0415 for 96 and 120 h. The numbers used to generate stacked graphs were obtained by analysis shown in (**B**) using the FACS data obtained from the BrdU incorporation assay coupled with 7-AAD staining and analyzed by FlowJo X software 10.0.7r2. (**D**) T8ML-1 cells were treated with either DMSO (vehicle) or DCZ0415 at indicated concentrations and counted using Trypan Blue exclusion dye. Bar graphs represent the total cell counts of live cells after 48 h (blue) or 72 h (red) of continuous drug treatment. Each data point is the average of three measurements taken in parallel and bar graphs represent ±SEM. (**E**) Immunoblot analysis of Cyclin B1 and Cyclin D1 protein levels in the T8ML-1 cell line treated with either DMSO (vehicle) or 10 uM DCZ0415 harvested and analyzed after 48 and 72 h of continuous drug treatment. HSC70 served as a loading control.

To test if targeting TRIP13 may have therapeutic potential, we next treated lymphoma cell lines with DCZ0415, a TRIP13-specific inhibitor [40]. A significant reduction in cellular proliferation was observed already after 4 days of the treatment with drug concentrations ranging from 1 µM to 10 µM. A 15-day continuous treatment of T8ML-1 cells severely reduced viability at all concentrations, especially at 5 µM and 10 µM of DCZ0415 (Figure 8A). Like with shRNA-mediated *TRIP13* downregulation, the treatment with the DCZ0415 inhibitor impaired cell proliferation by reducing BrdU incorporation, inducing G2-M arrest, and cell death (Figure 8B,C). The treatment of T8ML-1 cells with higher amounts of DCZ0415 (10–25 µM) showed dose-dependent cell death with EC50 values of 10 µM and 5.5 µM upon 48- and 72-h treatment, respectively (Figure 8D and Figure S27). Consistently with G2-M arrest observed at lower concentrations, Cyclin B1 level was elevated upon the drug treatment, whereas G1-S Cyclin D1 was relatively unchanged (Figure 8E and Figure S28).

Altogether, our data show that targeting TRIP13 may be beneficial in treating PTCL.

## 3. Discussion

The goal of our studies was to better understand the molecular landscape of PTCL with a focus on recurrent molecular changes and identification of putative genes driving lymphomagenesis. Using high-resolution methylation of 7 PTCLs and gene expression profiling of 10 PTCLs coupled with bioinformatic analysis of previously published data and functional studies, we made several interesting observations in this study.

First, hypomethylation of the PTCL genome is more frequent than hypermethylation, regardless of the genomic elements or tumor types. These data are consistent with the numerous studies in mouse models that demonstrated that hypomethylation in T–cell malignancies is more frequent than hypermethylation [17,20,41]. Furthermore, methylation patterns in tumors are heterogonous with large differences and only a few common features. Some studies in hematological malignancies found tumor methylation patterns to have little to no cancer specificity. For example, based on the profiling of chronic lymphocytic leukemia (CLL), it was proposed that tumor patterns are reflections of the methylation status of the cell of origin, rather than being truly cancer-specific because most previously reported tumor-specific methylation events are normally present in nonmalignant B–cells [42]. Similarly, recently identified DNA methylation patterns of ALCL were found to be similar to thymic progenitor cells [24]. Due to the tremendous methylation heterogeneity that we observed across PTCLs, the possibility that PTCLs reflect methylation patterns of the cell of origin appears unlikely. Rather, methylation differences between normal and malignant T–cells appeared to be a consequence of at least three molecular events that we observed with high-frequency—downregulation of DNMT3A and TET1 on the transcript level, and upregulation of TCL1 protein. The decrease in de novo and possible maintenance functions of DNMT3A and DNMT3B coupled with TCL1 inhibition of DNMTs may be responsible for hypomethylation, whereas downregulation of TET1 may have resulted in hypermethylation events. Thus, these molecular changes are likely responsible for the ‘Core PTCL Methylation Signature’ consisting of 767 hypo- and 567 hypermethylated DMRs that were observed across various genomic elements in all seven analyzed PTCLs. This signature had no overlap with the 12 gene signature (hyper—*BCL11B*, *CD5*, *CXCR6*, *GIMAP7*, *LTA*, *SEPT9*, *UBAC2*, *UXS1*); hypo—*ADARB1*, *NFIC*, *NR1H3*, *ST3GAL3*) observed in hepatosplenic T–cell lymphoma [25]. Interestingly, we observed hypermethylation of *LCK*, previously shown to be hypermethylated and repressed in ALCL and PTCL samples [24]. Although not part of the ‘Core PTCL Methylation Signature’, we also observed hypermethylation of *LEF1* and *TCF7*, but not *BCL11B* reported previously in PTCLs [24]. Thus, *LCK* promoter hypermethylation and, to a lesser degree, *LEF1* and *TCF7* belong to the most frequent molecular changes characterizing PTCLs. In addition to methylation signatures, we also identified the ‘Core PTCL Expression Signature’ consisting of 231 genes that were upregulated and 91 genes that were downregulated in all 10 tested tumors. Not surprisingly, at least 48 genes in this signature were associated with the cell cycle. Additional genes in the signature were related to complement activation, blood coagulation, and Notch pathway signaling. Several genes of the ‘Core PTCL Expression Signature’ are less characterized but their recurrent deregulation suggests a potential association with tumor development and have the potential to serve as biomarkers. Among them is *TEDC2* (also known as *C16orf59*) whose increased expression is associated with poor prognosis of lung adenocarcinoma [43]. Overexpression of *ZWINT* predicts poor prognosis and promotes the proliferation of hepatocellular carcinoma [44]. Increased *C10orf10* levels (DEPP Autophagy Regulator 1, *DEPP1*) correlated with the shorter survival time of patients with primary gliomas [45].

Second, we identified recurrent methylation events in promoters that were associated with the changes in gene expression. Namely, 39 genes upregulated in PTCL whose promoters were hypomethylated, and 56 genes repressed in tumors whose promoters were hypermethylated. Importantly, several oncogenes and tumor suppressor genes were identified in these signatures. For example, the hypomethylated and overexpressed gene *UHRF1* has oncogenic functions promoting tumorigenesis through the silencing of DNA repair genes and inhibiting apoptosis in various cancers [32]. *UHRF1* is overexpressed in T–cell ALL and its knockdown reduces c-MYC expression and viability in these malignancies [33]. Other genes hypomethylated and overexpressed in PTCL—*RAB13*, *MPZL1*, *CDK14*—were implicated in tumor progression of several different tumors including B-cell lymphomas, and glioblastomas, lung and ovarian [34,35,46,47]. Major tumor suppressors are among genes that we detected to be hypermethylated and silenced in PTCL, e.g., *ATM*, which plays a critical role in DNA repair and whose functions are often compromised in PTCL through acquired genetic alterations [48]. Another hypermethylated and silenced gene in PTCL—serine/threonine-protein kinase 4 (*STK4*)—regulates cell differentiation and apoptosis and is the tumor suppressor in hepatocellular carcinoma, breast cancer, and lymphoma [49]. We also detected somewhat puzzling hypermethylation and silencing of genes that would be predicted to be oncogenes, rather than tumor suppressors, such as *SF3B1*, *LCK*, *FOXP1*, and *FYN* [50,51]. Given the complexities of gene functions and the large-scale-deregulation of signaling networks in cancer, it is not surprising that some genes normally promoting tumorigenesis would be silenced. Alternatively, their effects on tumorigenesis would be context-dependent. For example, the loss of proto-oncogene *LCK* accelerates CLL development in mice, suggesting the tumor suppressor role for this gene [52]. The specific roles of genes whose expression changes coincided with methylation alterations remains to be worked out. However, our data strongly suggests that both DNA hypo- and hypermethylation affect the expression of genes that are important in various aspects of T–cell tumorigenesis and at least some of them are likely to contribute to PTCL development.

Third, we found that genetic and pharmacological inhibition of TRIP13 impaired the proliferation of T–cell malignant cell lines by inducing G2-M arrest and apoptosis. Interestingly, we identified mouse *TRIP13* to be hypomethylated and overexpressed in mouse T–cell lymphomas [21]. Our findings collectively underscore the critical role of DNA methylation in regulating *TRIP13*, as well as its significant involvement in the development and/or maintenance of TCL. This positions *TRIP13* as a potential key oncogene in PTCL. Reinforcing this hypothesis, similar conclusions have been drawn from research conducted on various solid tumors, where the influence of *TRIP13* on cancer progression has been similarly observed. For example, *TRIP13* is highly expressed in glioblastoma and its inhibition impaired the proliferation, migration, and invasion of tumor cells by regulating c-MYC stability [53]. TRIP13 promotes the metastasis of colorectal cancer and its inhibition decreased cell proliferation in vitro and tumor formation in vivo of colorectal carcinoma cell lines [40,54].

Our functional assays demonstrate that promoter hypomethylation is important even in human lymphomagenesis, at least in its maintenance. Whether it plays a role in initiation and progression remains to be tested.

It is important to note certain limitations in our study. Although our use of WGBS allowed for a more comprehensive analysis of the methylation landscape of PTCLs than previous studies [24,25], our research was limited to seven samples. This limitation might impact the generalizability of our findings in the broader context of PTCL research. Furthermore, while we performed RNA-seq gene-expression analysis on 10 PTCL samples, only seven of those lymphomas were profiled for DNA methylation alterations due to the unavailability of DNA. Altogether, this limited our ability to address tumor-specific differences between ALCL (T1–4), AITL (T5), and PTCL-NOS (T5–10). Thus, by and large, we did not attempt to dissect the molecular differences within tumor subtypes because such analysis would result in a large number of false-positive and negative alterations and therefore provide substantially skewed views of molecular landscapes. Rather, our focus was on methylation and gene expression landscapes of PTCLs analyzed as one single tumor entity in an attempt to identify potential molecular events involved in the pathogenesis of these malignancies. In one analysis attempting to classify PTCL-NOS into *TBX21* or *GATA3* subgroups, we were unable to do so either using their gene expression values or readout of their target genes. While this might be due to the limited sample size, it is also possible that such classification of PTCL-NOS, which was primarily based on microarray results, may not be easily seen by RNA-seq analysis or be less pronounced than previously thought.

Our future study will also address the relationships of methylation and gene expression changes observed in this study. However, given the large number of genes with demonstrated biological effects in T–cell biology, our study clearly points to a causative relationship of deregulated methylation in PTCL development.

## 4. Materials and Methods

### 4.1. Clinical Samples and Data Sources

The study included in total two normal human purified CD4+ T–cells (84200-1.0/13122) and normal human purified CD8+ T–cells (84300-1.0/13083) obtained from Precision Medicine. These samples were used for methylation analysis by WGBS and gene expression analysis by RNA-seq. We also used seven frozen PTCL tissue samples and one normal lymph node sample obtained from the NCI Cooperative Human Tissue Network (CHTN). Other investigators may have received samples from the same tissue specimens. PTCL samples were used for the isolation of DNA (for WGBS), RNA (RNA-seq and real-time qRT-PCR), and proteins (immunoblots). The frozen lymph node was used for protein isolation and served as a control for immunoblots. An additional three total RNA samples isolated from PTCL tumors and one paired DNA sample were acquired from Origene (Rockville, MD, USA). Methylation and gene expression analyses were performed on paired DNA-RNA samples except for two RNA samples for which DNA or tissue was not available. The Institutional Review Board of the University of Nebraska Medical Center approved the use of these samples. The sample sets are described in this section and in Figures S1 and S3. For data analysis, we utilized several data sets available online. For the methylation analysis—in addition to normal human T–cell controls generated in this study—we also used publicly available WGBS data on CD3+ and CD4+ T–cells obtained from GEO [GSM1186660, GSM2103005, GSM2103006, GSM2103007] [55]. For gene expression analysis, publicly available data for human ALCL (SRA accession numbers SRR1522989, SRR1522990, SRR1522992, SRR1522994, SRR1522996, SRR1522997, SRR1522998, SRR1522999, SRR1523000, SRR1523001, SRR1523002, SRR1523003, SRR1523004, SRR1523005, SRR1523006, SRR1523007, SRR1523008, SRR1523009, SRR1523010, SRR1523011, SRR1523012), ATLL—GSE143986 (SRR10918774, SRR10918775, SRR10918776, SRR10918777, SRR10918778, SRR10918779, SRR10918780, SRR10918781) [56] NKTCL (SRR1648322, SRR1648328, SRR1648327, SRR1648323, SRR1648332, SRR1648329, SRR1648315, SRR1648333, SRR1648324, SRR1648318, SRR1648326, SRR1648330, SRR1648321, SRR1648331, SRR1648325) and TLBL -GSE109231 (SRR6495843, SRR6495840, SRR6495836, SRR6495842, SRR6495844, SRR6495835, SRR6495837, and SRR6495841 [57] were used. (https://bmccancer.biomedcentral.com/articles/10.1186/s12885-018-4304-y, accessed on 16 April 2018) [55] (Supplementary Materials S7).

### 4.2. Plasmid DNA Constructs

Lentiviral vectors pLV[shRNA]-mCherry-U6>scrambled [shRNA#1] with a target sequence of CCTAAGGTTAAGTCGCCCTCG, pLV[shRNA]-mCherry-U6>hTRIP13 [shRNA#1] with a target sequence of GCTACTCAACAGACATAATAT, pLV[shRNA]-mCherry-U6>hTRIP13[shRNA#2], with a target sequence of GATGAAGTGTCAGATCATATA, pLV-EF1A-mCherry and pLV-EF1A hTRIP13 -3XFLAG- P2A-mCherry were obtained from VectorBuilder.

### 4.3. Cell Lines and Lentiviral Production

Human peripheral T–cell lymphoma cell line T8ML-1 was a generous gift from Dr. Hiroshi Fujiwara (Ehime University, Ehime, Japan). Human acute T–cell leukemia cell line JURKAT (#TIB-152) was obtained from the American Type Culture Collection (ATCC, Gaithersburg, MD, USA). Lenti-XTM 293T Cell Line was purchased from Takara (#632180, Clontech, San Jose, CA, USA). The cell lines were periodically checked for karyotype and tested for mycoplasma contamination. Only early passages were used.

Cells were maintained in DMEM or RPMI 1640 (Invitrogen, Carlsbad, CA, USA) containing 10% fetal bovine serum. Cell lines were cultured at 37 °C in a humidified 5% CO_2_ atmosphere and were passaged according to recommendations.

To generate lentiviruses, 293T cells were seeded in a 100 mm tissue culture plate to obtain ~80–90% confluence and transfected with plasmid DNA and packaging plasmids psPAX2 and pMD2.G at a ratio 1:0.65:0.35 using 70 μg of polyethylenimine (PEI) (Polysciences, Warrington, PA, USA). Viruses were collected 48–96 h post-transfection. Transduction was performed as described previously [17] using the Polybrene Infection/Transfection Reagent (Sigma, St. Louis, MO, USA). The efficiency of transduction was determined by measuring the percentage of mCherry-positive cells by FACS.

### 4.4. FACS, BrdU Incorporation, and Apoptosis Assays

For shRNA experiments, the growth of T8ML-1 or JURKAT cells transduced with lentiviruses was monitored by FACS over 2–20 days. The maximum percentage of mCherry-expressing cells was typically observed 72 h post-transduction. In some experiments, all subsequent mCherry data points were normalized to this time point and expressed as a relative percentage of the initial time point. EGFP was measured periodically by flow cytometry on the LSRII available at the University of Nebraska flow cytometry core facility. In JURKAT cells, where we achieved +99% transduction efficiency, the growth of cells was evaluated by Trypan blue staining and cell counting at different time points. Viability was also evaluated by scoring percentages of cells in the “Live gate” of FACS diagrams obtained using cells at different timepoints. For in vitro BrdU labeling, BrdU at a concentration of 0.01 mM was added 110 min before harvests of cells. BrdU-positive cells were quantified using anti-BrdU-FITC (BrdU- Flow Kit, BD Biosciences PharMingen, San Diego, CA, USA) as described by the manufacturer. For cell cycle analysis, 7-ADD was added to the samples. For analysis of apoptosis, cells were stained with allophycocyanin-conjugated annexin V antibody and analyzed by FACS according to the manufacturer’s recommendations (eBioscience, San Diego, CA, USA). FACS analysis was performed using the BD Accuri C6 Plus Flow Cytometer.

### 4.5. WGBS and Bioinformatics Analysis

Genomic DNA from PTCL samples was isolated using the QIAamp DNA Mini Kit (Qiagen, Germantown, MD, USA).

The WGBS libraries using DNA from normal human CD4+ and CD8+ purified T–cells and seven PTCLs were prepared and sequenced on an Illumina NovaSeq6000 sequencer using 150 bp long paired-end reads by Novogene, USA. Quality checks, trimming, filtering, alignment of reads to the UCSC hg38 reference genome, and methylation calling were performed with Bismark software 0.22.1 [58]. Only CpG sites with a minimum sequencing depth 5x were included in the analysis. Differential methylation was calculated by comparison of individual tumors to the control consisting of methylation data obtained from five normal human T–cells—CD3, CD4_1, CD4_2, CD4_3, and CD8—averaged out by Metilene.

Differentially methylated regions (DMRs) were determined by Metilene [59] and defined based on the average of a minimum of three consecutive DMCs with a methylation change of ≥10% in the same direction with *p* values < 0.05 (as determined by the MWU test). The maximal base pair cut-off for a distance between consecutive DMCs in the DMR was set to 100 bp. Annotation of methylated CpGs and DMRs to promoters, gene bodies, enhancers, and repeats was performed using bedtools intersect. Chromosomal coordinates of TSS, gene bodies, and repeats were acquired from the USCS Table browser. Enhancer coordinates identified in CD4+CD8+ cells and thymus cells were obtained from Enhancer atlas [60]. Promoter was defined as 1500 bp upstream to 500 bp downstream of the TSS.

Methylation scores were visualized with the Integrated Genome Browser (IGB) [61]. Scatter plots of the methylation score were generated in RStudio v1.1.4.6 using package gplots. Genome-wide Pearson correlation analysis of CpG sites was performed using deepTools package multi bigwig summary and plot correlation [62].

### 4.6. Immunoblotting

Frozen tissue was cut on a glass plate on dry ice and 20–50 mg of tissue was placed into a round bottom Eppendorf tube. Then, 20 µL/mg of ice-cold RIPA buffer (50 mM Tris-HCl (pH 7.4), 150 mM NaCl, 1 mM EDTA, 1% NP-40, 1% Na-deoxycholate, 0.1% SDS, sterile-filtered) along with protease inhibitor cocktail and sodium orthovanadate was added and the tissue was homogenized with an electric homogenizer on ice. After 20 min incubation on ice, samples were centrifuged at 13,000× *g* for 20 min at 4 °C. The supernatant containing the soluble protein was collected into a new tube kept on the ice. Immunoblots were performed as previously described [17], using the following antibodies detecting: TRIP13 (cat.# TA809737S, Origene; dilution 1:1000), Cyclin B1 (cat.# 4138, Cell Signaling; dilution 1:1000), Cyclin D1 (cat.# 2978, Cell Signaling; dilution 1:1000), Cdc25A (cat.# SC-97, Santa Cruz; dilution 1:500) and HSC70 (cat.# SC-7298, Santa Cruz; dilution 1:10,000). Relative densitometric values of protein levels were calculated using ImageJ software 1.54d.

### 4.7. RNA Isolation, RNA-Seq, and Bioinformatics Analysis

Total RNA from frozen tumor tissues was isolated using the TRIzol reagent (Invitrogen) and repurified using an RNAeasy kit (Qiagen). Library generation and sequencing on the NovaSeq 6000 platform using paired-end 150 bp runs was performed by Novogene, USA. Tissue samples from seven peripheral T–cell lymphomas were provided by the NCI Cooperative Human Tissue Network (CHTN). Other investigators may have received samples from the same tissue specimens. Three total RNA samples isolated from PTCL tumors were acquired from Origene (Rockville, MD, USA). The Institutional Review Board of the University of Nebraska Medical Center approved the use of these samples. The sample description is summarized in Supplementary Figure S2.

Publicly available data for human ALCL, ATLL, NKTCL, and TLBL were obtained from GEO [55] and SRR (accession numbers listed in Supplementary Materials S7). Trimmed sequencing data were first aligned to *Homo sapiens* UCSC hg38 reference genome using STAR aligner. RNA-seq data with minimum mapped quality 50 were quantified using the RNA-seq quantification pipeline in SeqMonk software 1.47.2ESeq2 was used to calculate differential expression. For differentially expressed genes, only genes with a fold change ≥ 1.5 and a *p* value < 0.05 were considered significant. Ingenuity pathway analysis (Qiagen) [63] was used to analyze activated and decreased core signaling pathways for differentially expressed genes. Activated and inhibited pathways (Z-score > 1.5, *p* < 0.05) identified in individual PTCLs are shown in Figure 3B. The top subcategories obtained in Physiological System, Development, and Functions are displayed (*p* < 0.05, for all subcategories).

### 4.8. TRIP13 Drug Treatment, Cell Counting, and Molecular Assays

DCZ0415, an inhibitor of TRIP13, was obtained from (MedChem, Cat. #: HY-130603, Monmouth Junction, NJ, USA) and dissolved in DMSO. T8ML-1 cells were subjected to treatments with either DMSO (serving as the vehicle control) or the DCZ0415 at concentrations ranging from 1 to 25 µM per 1 mL of culture. The cell viability was assessed using Trypan Blue exclusion dye cell counts at different time points after the drug addition. EC50 values were determined by assessing the drug concentration that corresponds to 50% cell viability and were analyzed by AAT Bioquest Software. For the analysis of TRIP13 protein levels upon the treatment, 1 × 10^8^ T8ML-1 cells were treated with DMSO or 20 µM DCZ0415 per 1 mL of culture, and protein lysates were made similarly as described above. BrdU assays and apoptosis were performed as described above.

### 4.9. Real-Time qRT-PCR

Two micrograms of RNA were reverse transcribed with the SuperScript III Reverse transcriptase (Thermo Fisher, Waltham, MA, USA) using oligo (dT) primers. Real-time qRT-PCR was performed with the iQ™ SYBR^®^ Green Supermix (Bio-Rad, Hercules, CA, USA) on a CFX96 Touch™ Real-Time PCR Detection System (Bio-Rad). Fast PCR cycling conditions were used (95 °C for 3 min, 40 cycles (95 °C for 10 s, 58–63.5 °C for 30 s)), followed by a dissociation curve analysis. All qRT-PCR measurements were performed in duplicate reactions and normalized to the expression of the housekeeping gene (*β-actin*). In parallel, no-RT controls were amplified to rule out the presence of contaminating genomic DNA (Supplementary Materials S8).

### 4.10. Statistical Analysis

The statistical significance of means ±SEM was evaluated using the two-tailed Student’s *t*-test. For all statistical analyses *p* values < 0.05 were considered significant.

## Data Availability

All relevant data are available from the corresponding author upon reasonable request. The WGBS and RNA-seq are deposited at the NCBI Gene Expression Omnibus database [wgbs_accesion, GSE154451] [55].

## Supplementary Materials

The following supporting information can be downloaded at: https://www.mdpi.com/article/10.3390/epigenomes8030032/s1, Figure S1: Summary of the coverage obtained from the Whole Genome Bisulfite sequencing of normal T-cell samples; Figure S2: Tumor classification and summary of pathological information for PTCLs used in this study; Figure S3: Summary of the coverage obtained from the Whole Genome Bisulfite sequencing of indicated Peripheral T-cell lymphomas (PTCLs); Figure S4: The relative ratios of hypomethylated to hypermethylated DMRS in the ‘Core PTCL Methylation Signature’ across genomic elements calculated from the data presented in Figure 2H and Supporting Information 3; Figure S5: Heat map presentation and the numbers of hypomethylated and hypermethylated DMRs distributed among indicated genomic elements detected with the frequency of 7/7 tumors; Figure S6: Percentage of methylation at individual CGs in UCK2 and TXK loci as visualized by IGB software; Figure S7: The TOP 10 up and downregulated genes in the ‘Core PTCL Gene Expression Signature’; Figure S8: Pathway enrichment analysis of upregulated genes in the ‘Core PTCL Gene Expression Signature’ after the removal of 48 cell cycle-related genes; Figure S9: ChEA enrichment analysis of 231 upregulated genes in the ‘Core PTCL Gene Expression Signature’; Figure S10: Pathway enrichment analysis of downregulated genes in the ‘Core PTCL Gene Expression Signature’; Figure S11: ChEA enrichment analysis of 91 down-regulated genes in the ‘Core PTCL Gene Expression Signature’; Figure S12: Heat map presenting fold change expression values of a subset of genes reported as PTCL-TBX21 and PTCL-GATA3 signatures (6); Figure S13: Gene expression of TET2, TET3, and TCL1B in human PTCLs and control T-cells as analyzed by RNA-seq; Figure S14: Comparative expression of TET1, TET3, and TCL1B in various T-cell lymphomas and control T-cells; Figure S15: Overlap between methylation and expression of repetitive elements; Figure S16: Profile of hypomethylated and overexpressed genes in 5 out of 7 tumors; Figure S17: Pathway enrichment analysis of hypomethylated and overexpressed genes in tumors using EnrichR and WikiPathways; Figure S18: Profile of hypermethylated and downregulated genes in 5 out of 7 tumors; Figure S19: Pathway enrichment analysis of downregulated and hypermethylated genes in tumors using EnrichR and WikiPathways; Figure S20: Impact of gene knockouts on hematologic cell line proliferation using CRISPR screens from the DepMap database; Figure S21: Uncropped version of western blot analysis of TRIP13 protein expression in hematologic cell lines shown in Figure 6C; Figure S22: Uncropped version of western blot analysis of TRIP13 protein expression in hematologic cell lines shown in Figure 6C; Figure S23: Time-course analysis of mCherry expression in T8ML-1 cells post-transduction with TRIP13 shRNA-2 and scrambled control, as measured by FACS; Figure S24: Assesment of transduction efficiency using Fluorescence-Activated Cell Sorting (FACS); Figure S25: Uncropped version of western blot analysis of TRIP13 protein expression after shrna knockdown shown in Figure 7D; Figure S26: TRIP13 shRNA-1 and shRNA-2 downregulate TRIP 13 in JURKAT cells; Figure S27: DCZ0415 treatment of T8ML-1 cells impairs proliferation in vitro; Figure S28: Uncropped version of western blot analysis of cell cycle proteins after DCZ0415 treatment shown in Figure 8E. Supplementary Materials S1: Supporting Information for Figure 1C methylation status of 34,858 promoters in normal CD4 and CD8 by WGBS; Supplementary Materials S2: Supporting Information for Figure 1E methylation status of 19,253 promoters in normal CD4 and CD8 by WGBS and Figure 1E gene expression (FPKM values) of 19,253 promoters in normal CD4 and CD8 by RNA-seq; Supplementary Materials S3: Supporting Information for Figure 2H—PTCL methylation signature containing 767 hypomethylated DMRs present in all seven PTCLs and Figure 2H—PTCL methylation signature containing 567 hypermethylated DMRs present in all seven PTCLs; Supplementary Materials S4: Supporting Information for Figure 3A—Fold change expression values of differentially expressed genes in 10 human T-cell lymphomas; Supplementary Materials S5: Supporting Information for Figure 3C—Core gene expression signature (Fold change values) of 10 PTCL as determined by RNA-seq; Supplementary Materials S6: Supporting Information for Figure S16—Methylation profile of hypomethylated and overexpressed genes in 5 out of 7 tumors and for Supplementary Figure S18—Methylation profile of hypermethylated and downregulated genes in 5 out of 7 tumors Supplementary Materials S7: Publicly available data for gene expression analysis of human T-cell lymphomas; Supplementary Materials S8: Primer Sequences for qPCR.
