# Supplementary material for: Genome-Wide Methylation Profiling of Peripheral T–Cell Lymphomas Identifies TRIP13 as a Critical Driver of Tumor Proliferation and Survival
Source: Epigenomes. 2024 Aug 21;8(3):32. doi: 10.3390/epigenomes8030032 (PMC11348144; doi:10.3390/epigenomes8030032)
Supplement: Supplementary file 1 [file epigenomes-08-00032-s001.zip › epigenomes-3019185-supplementary/epigenomes-3019185-supplementary-1.pdf]

Supporting information for:

Genome-wide methylation profiling of Peripheral T-cell lymphomas identifies TRIP13 as a critical driver of tumor proliferation and survival

Pawel Nowialis[1*$], Julian Tobon[1*], Katarina Lopusna[1*&], Jana Opavska[1], Arshee Badar[1], Duo Chen[1], Reem Abdelghany[4], Gene Pozas[4], Jacob Fingeret[4], Emma Noel[5], Alberto Riva[2], Hiroshi Fujiwara[3], Alexander Ishov[1], Rene Opavsky[1,#]

[*]Equal contribution

Content:
Supporting figures S1-S28

	GEO	5x Cs
CD3	GSM1186660	38,585,864
CD4_86 (CD4_1)	GSM2103007	45,840,373
CD4_821 (CD4_2)	GSM2103005	38,584,229
CD4_822 (CD4_3)	GSM2103006	39,276,501
CD4	To be submitted	27,438,125
CD8	To be submitted	31,678,787

Figure S1. Summary of the coverage obtained from the Whole Genome Bisulfite sequencing of normal T-cell. samples. Samples CD4 and CD8 were profiled by WGBS in our studies whereas samples CD3,CD4_1, CD4_2 and CD4_3 were obtained from publicly available databases. The number of reads covering every cytosine in the genome by at least 5 reads is shown.

PTCL#	PATHOLOGY
1	CD4+ Anaplastic large cell lymphoma, ALK-1 neg, 80-90% ki67+
2	CD4+ Anaplastic large cell lymphoma, ALK-1 neg
3	CD8+ Atypical large cell lymphoma. Express B- and T-cell markers
4	CD4+ Anaplastic large cell lymphoma, ALK-pos
5	CD4+ Angioimmunoblastic T-cell lymphoma, 84% pure
6	CD3+ PTCL-NOS lymphoma (CD4 and CD8 unknown), 50% ki67+
7	CD8+ PTCL-NOS lymphoma, 93% pure
8	PTCL-NOS lymphoma. CD3 ~ Leu4 ~ UCHT1 by FC, Positive; Chromosome structure, Other chromosome structure; CD19 ~ B4 ~ Leu12 by IHC, Positive; Kappa light chain by IHC, Positive; CD8 ~ Leu2a ~ T8 by IHC, Positive; Lambda light chain by IHC, Positive
9	PTCL-NOS lymphoma. CD3 ~ Leu4 ~ UCHT1 by IHC, Positive; CD7 ~ Leu9 by FC, Positive; CD4 ~ Leu3a by IHC, Positive; CD7 ~ Leu9 by IHC, Positive; CD25 ~ Tac by IHC, Positive; CD20 ~ B1 ~ Leu16 by IHC, Positive
10	PTCL-NOS lymphoma. CD3 ~ Leu4 ~ UCHT1 by FC, Positive; CD19 ~ B4 ~ Leu12 by IHC, Negative; CD22 ~ Leu14 by IHC, Negative; CD33 ~ My9 by IHC, Negative; Kappa light chain by IHC, Negative; CD8 ~ Leu2a ~ T8 by IHC, Negative

Figure S2. Tumor classification and summary of pathological information for PTCLs used in this study.

tumors	5x Cs	overlap with control
1	18,965,776	9,840,048
2	24,208,809	10,483,032
3	16,555,643	8,735,585
4	24,911,908	12,297,899
5	17,584,638	8,716,849
6	24,889,740	10,661,540
8	17,539,642	8,814,322

Figure S3. Summary of the coverage obtained from the Whole Genome Bisulfite sequencing of indicated Peripheral T-cell lymphomas (PTCLs). The number of reads covering every cytosine in the genome by at least 5 reads as well as the number of cytosine overlapping controls is shown. 7,628,019 cytosines overlapped in all samples.

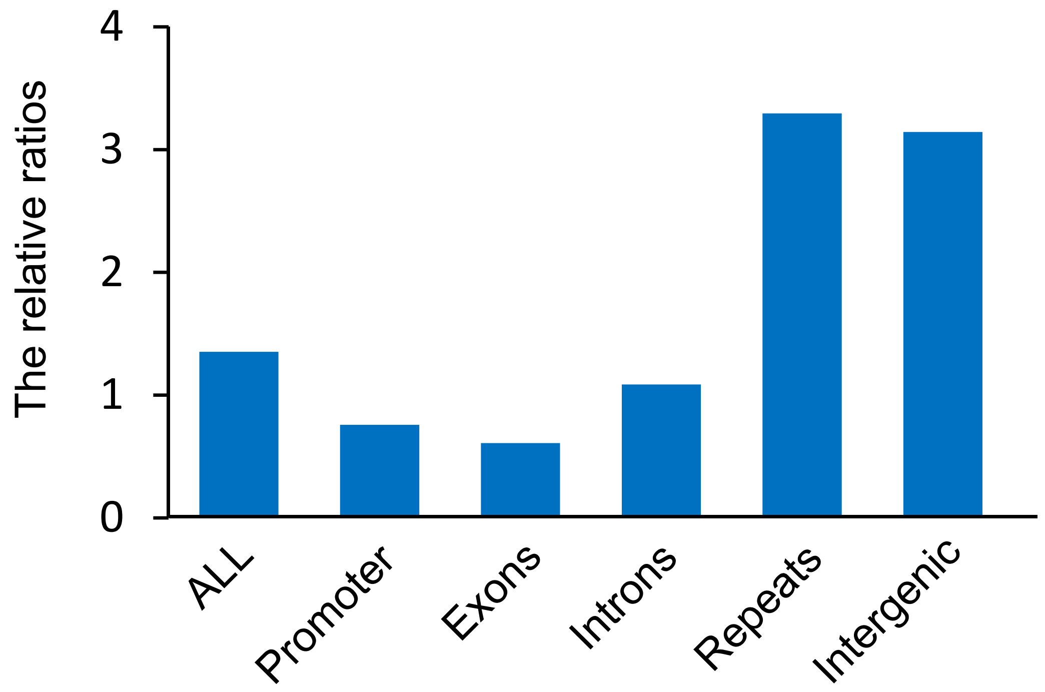

Figure S4. The relative ratios of hypomethylated to hypermethylated DMRS in the ‘Core PTCL Methylation Signature’ across genomic elements calculated from the data presented in Fig. 2H and Supporting Information 3.

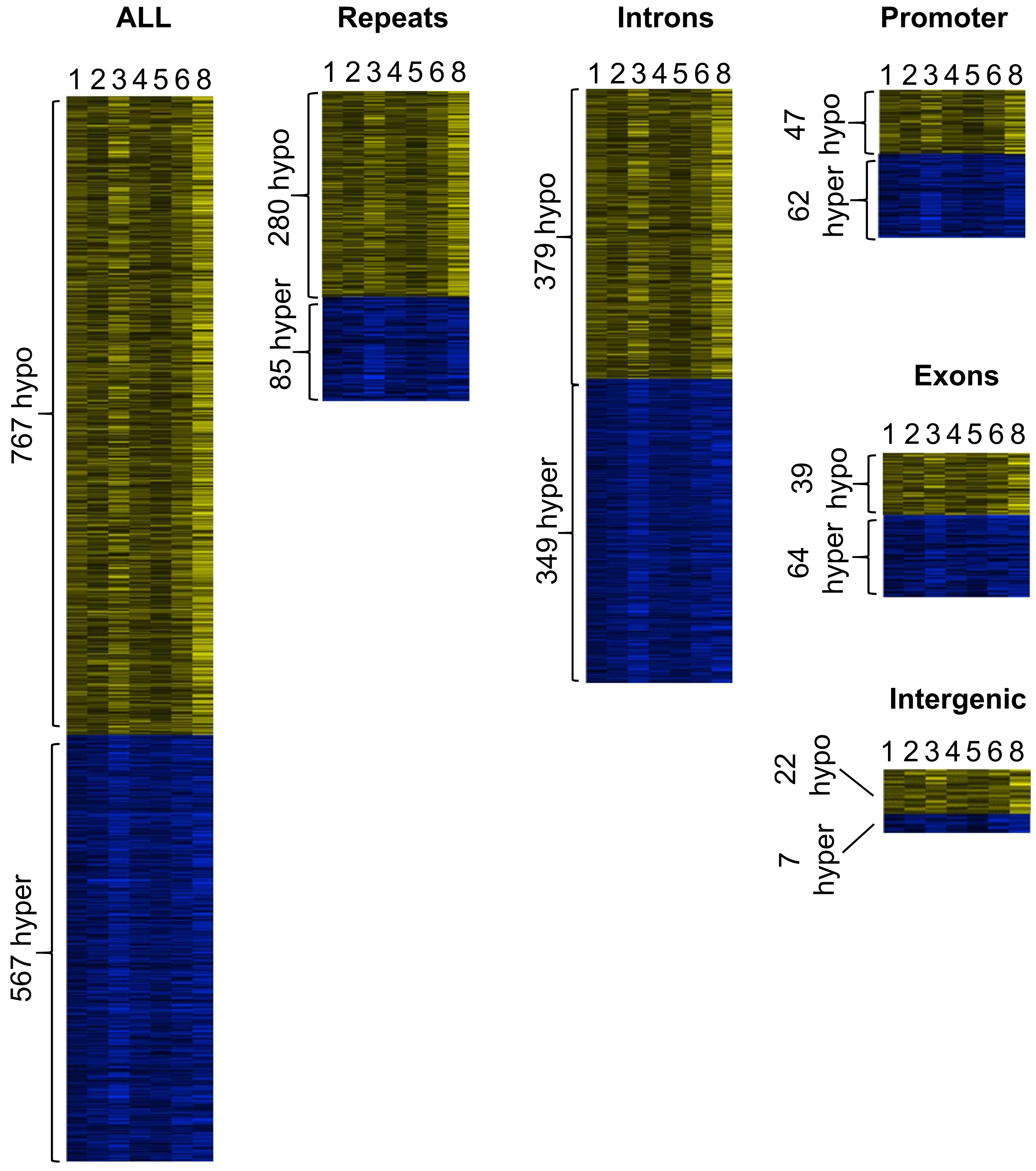

Figure S5. Heat map presentation and the numbers of hypomethylated and hypermethylated DMRs distributed among indicated genomic elements detected with the frequency of 7/7 tumors. The corresponding loci are listed in Supporting Information 3.

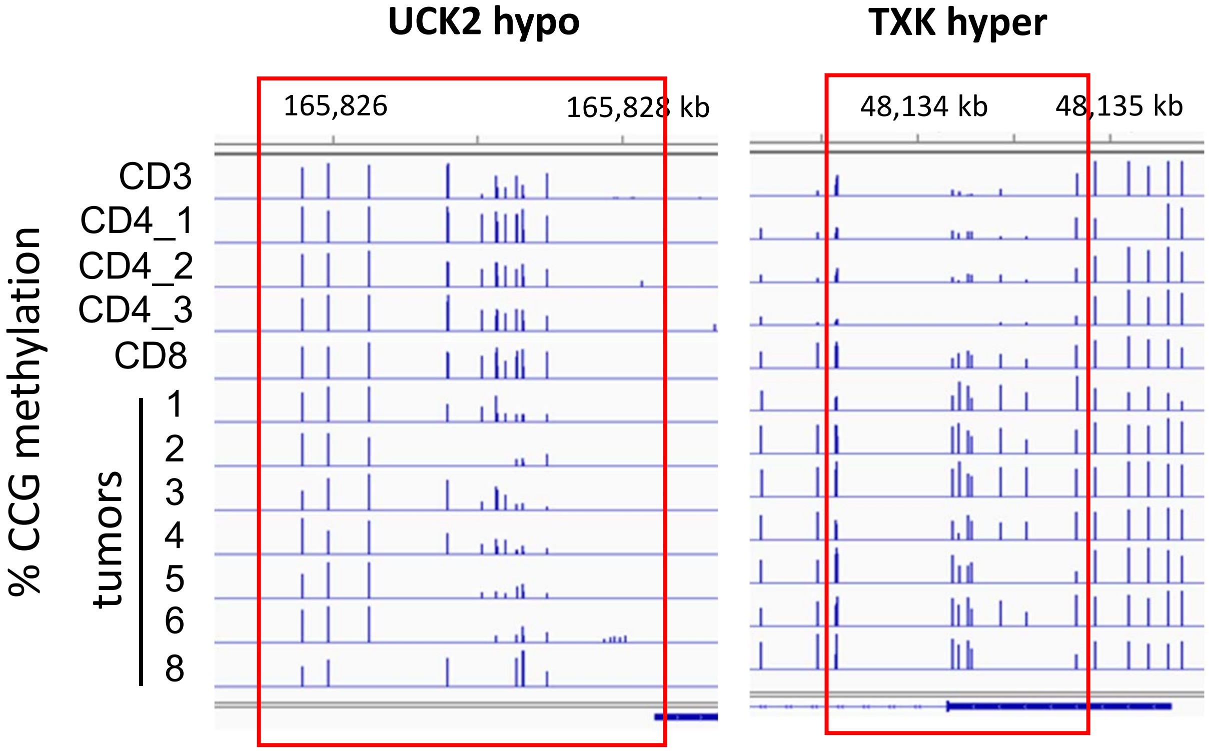

Figure S6. **Percentage of methylation at individual CGs in UCK2 and TXK loci as visualized by IGB software.**

	tumors									
	1	2	3	4	5	6	7	8	9	10
PLA2G2D	1190	783	89	1646	918	2537	134	171	2047	726
SLCO2B1	1120	494	129	1229	906	1209	350	401	830	846
ADAMDEC1	858	225	389	750	590	1459	57	98	1419	329
C1QB	1147	145	331	580	1390	994	170	445	706	232
RAMP3	306	842	122	620	375	458	694	130	1681	549
C1QC	1035	143	636	533	1017	337	215	399	679	252
GGT5	557	578	128	1638	511	351	220	59	771	233
TNFRSF11B	716	226	25	326	926	501	281	153	646	594
TDO2	206	338	109	328	1188	671	100	88	1134	52
KCNE4	241	159	51	761	448	453	150	43	1088	363
USP9Y	-3	-3	-190	-3	-1016	-10102	-240	-5	-10	-9
TTTY15	-3	-4	-124	-3	-440	-2187	-519	-3	-5	-8
UTY	-2	-3	-92	-2	-887	-1103	-174	-2	-4	-8
TENM1	-16	-17	-1409	-5	-5	-20	-4	-5	-24	-2
FGFBP2	-65	-169	-114	-67	-32	-53	-406	-228	-35	-98
CELF2-AS1	-9	-57	-136	-68	-10	-28	-10	-447	-80	-7
PDE3B	-6	-3	-558	-6	-13	-7	-2	-147	-3	-3
ITK	-10	-5	-665	-5	-4	-5	-3	-13	-3	-3
S1PR5	-19	-17	-51	-16	-12	-30	-42	-250	-27	-48
MAGEE1	-5	-2	-416	-5	-5	-6	-3	-26	-6	-4

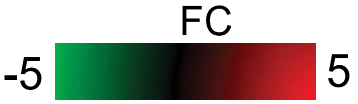

Figure S7. **The TOP 10 up and downregulated genes in the 'Core PTCL Gene Expression Signature'**. The numbers in the boxes represent a fold increase over the mean of normal T-cells (n = 4).

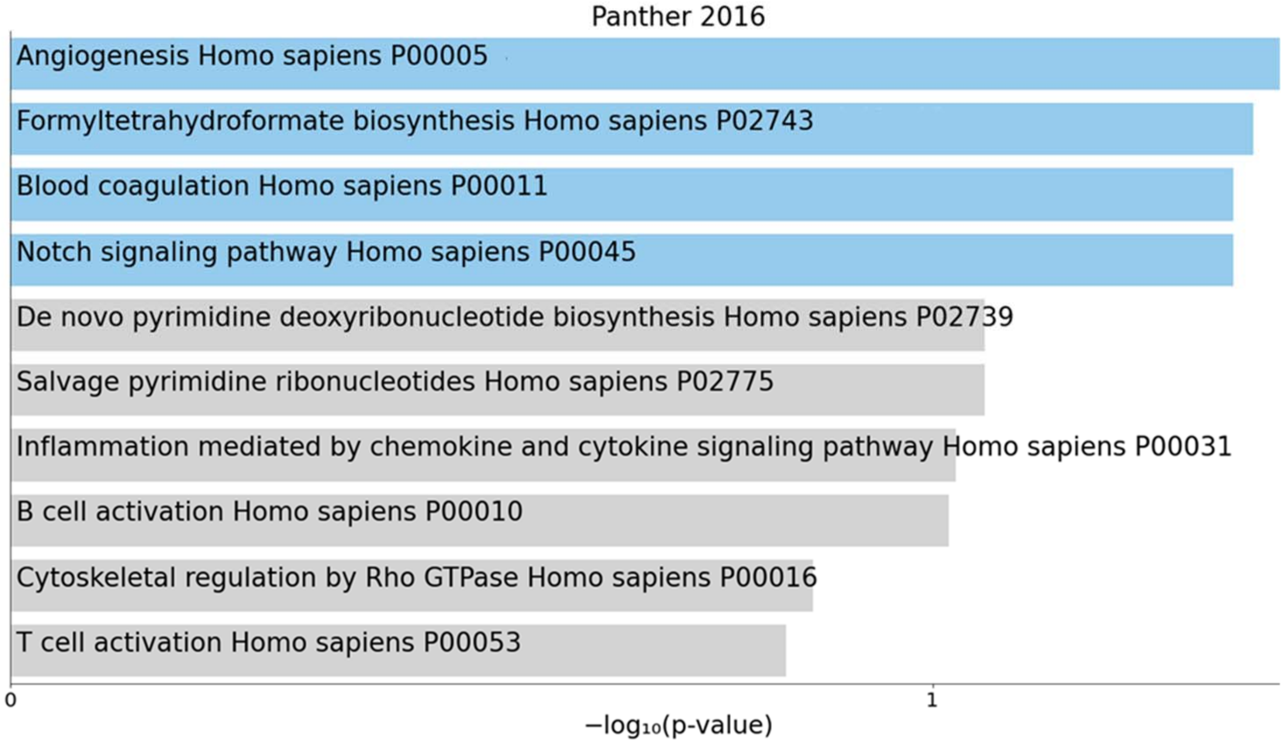

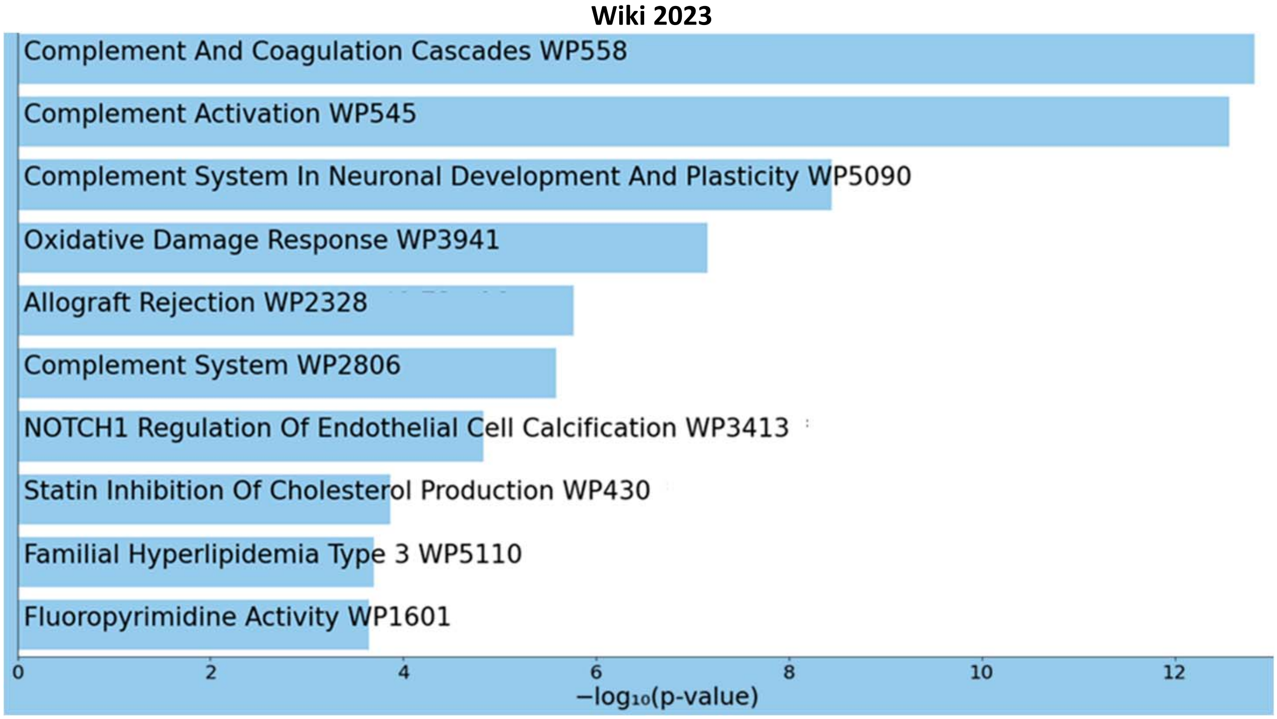

Figure S8. Pathway enrichment analysis of upregulated genes in the 'Core PTCL Gene Expression Signature' after the removal of 48 cell cycle-related genes. The 'core' gene signature consisting of 231 upregulated genes relative to the average of normal T-cells (n = 4) in all tested PTCLs (n = 10) as determined by RNA-seq data analysis was analyzed after the removal of 48 cell cycle-related genes by *Panther 2016* and *WikiPathway 2023*. The top signaling pathways are shown with a significance threshold of $p < 0.05$.

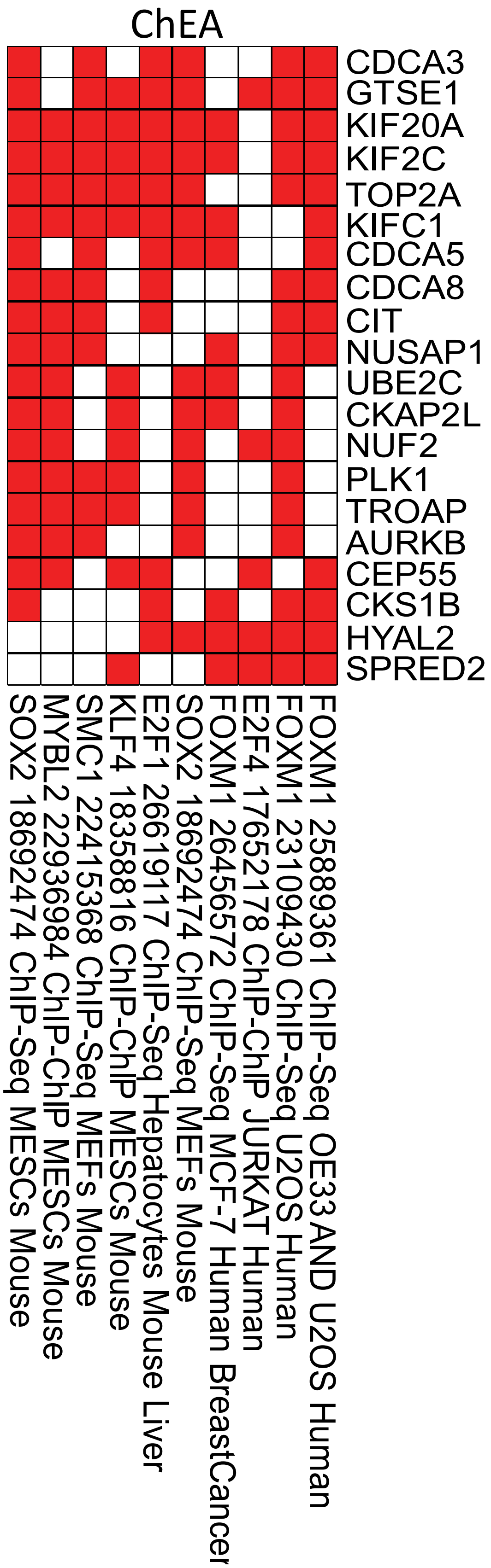

Figure S9. ChEA enrichment analysis of 231 upregulated genes in the 'Core PTCL Gene Expression Signature'. The data were analyzed by GO ChEA software and the top hits ChIP data sets are shown.

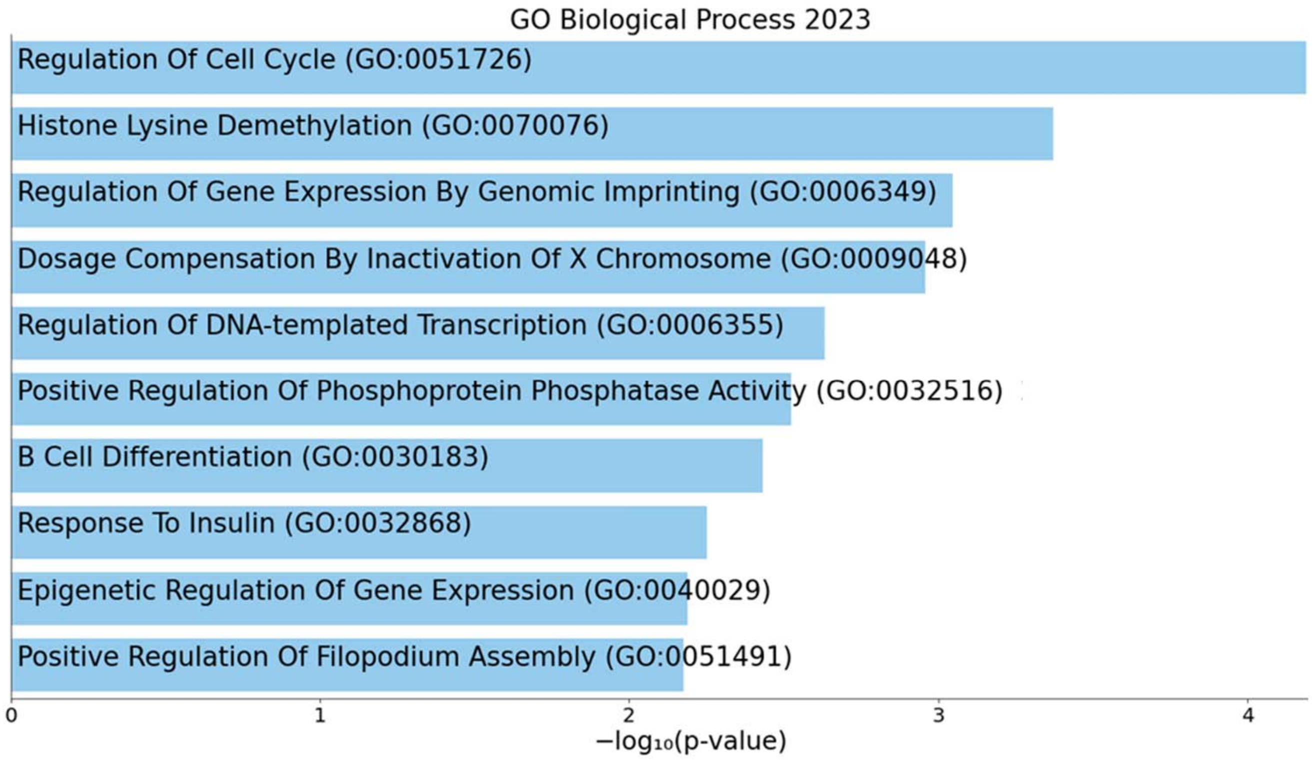

Figure S10. Pathway enrichment analysis of downregulated genes in the 'Core PTCL Gene Expression Signature'. The 'core' gene signature consisted of 91 down-regulated genes relative to the average of normal T-cells (n = 4) in all tested PTCLs (n = 10) as determined by RNA-seq data analysis. The data were analyzed by GO in the category "*Biological Processes*". The top signaling pathways are shown with a significance threshold of $p < 0.05$.

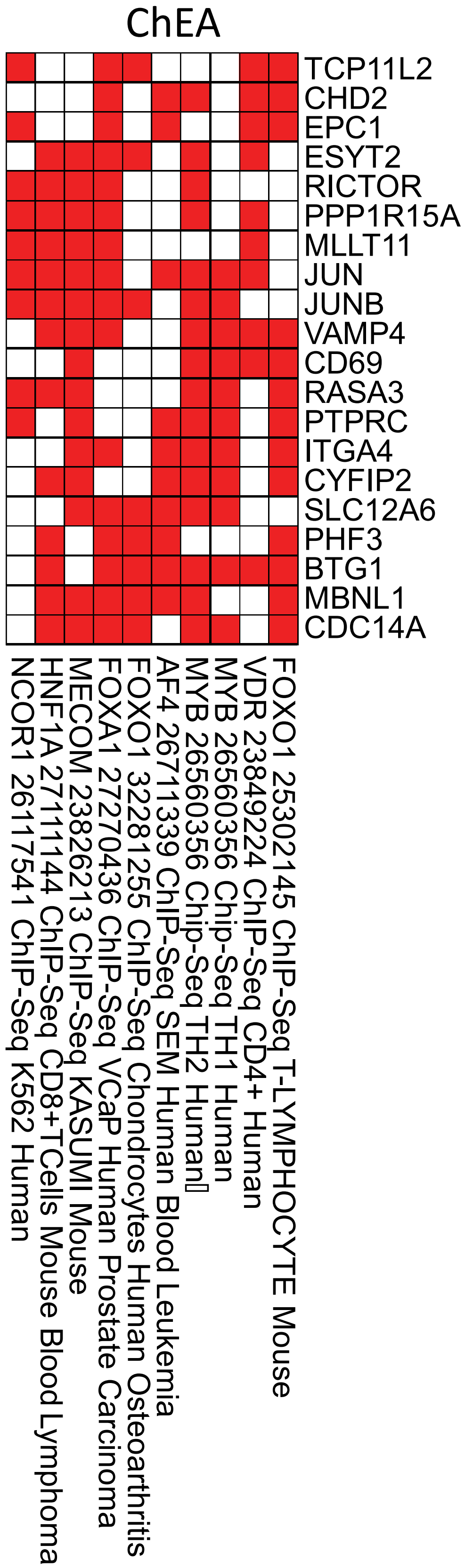

Figure S11. ChEA enrichment analysis of 91 down-regulated genes in the 'Core PTCL Gene Expression Signature'. The data were analyzed by GO ChEA software and the top hits ChIP data sets are shown.

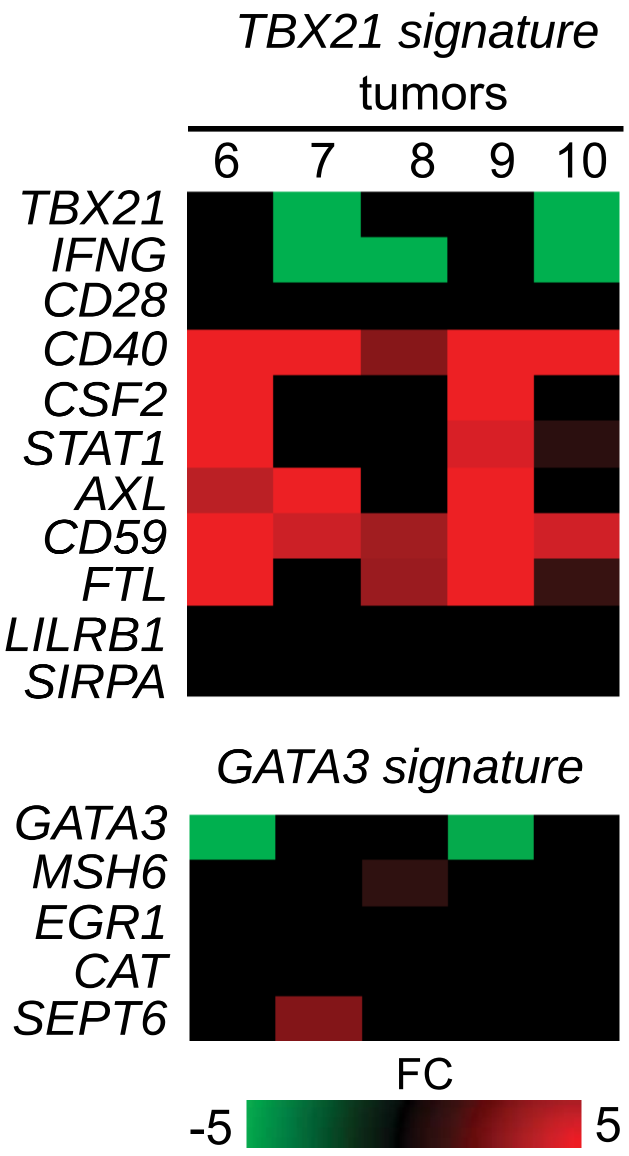

Figure S12. Heat map presenting fold change expression values of a subset of genes reported as PTCL-*TBX21* and PTCL-*GATA3* signatures (6). Presented tumors (#6-10) were classified as PTCL-NOS in pathological reports.

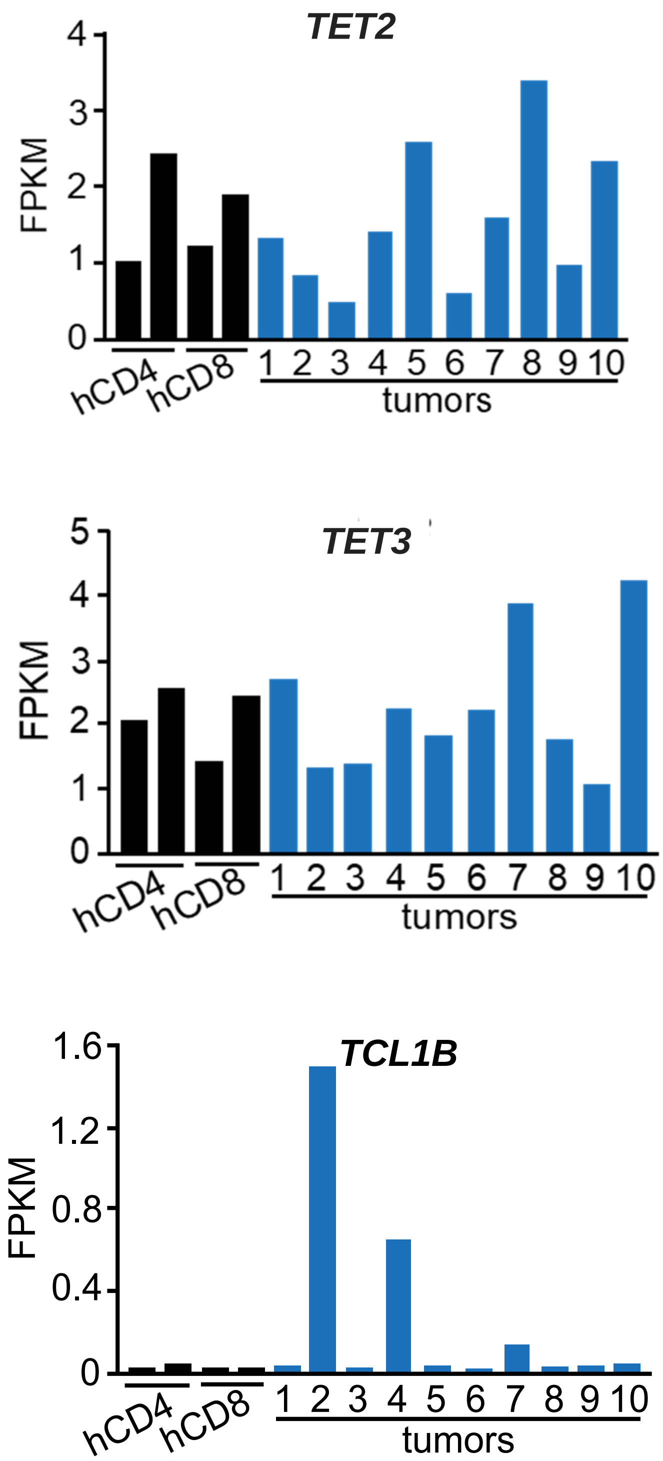

Figure S13. Gene expression of *TET2, TET3,* and *TCL1B* in human PTCLs and control T-cells as analyzed by RNA-seq. Statistically significant differences were calculated by comparison of individual tumors to averaged control T-cells (n = 4) *p < 0.05 (by DESeq2).

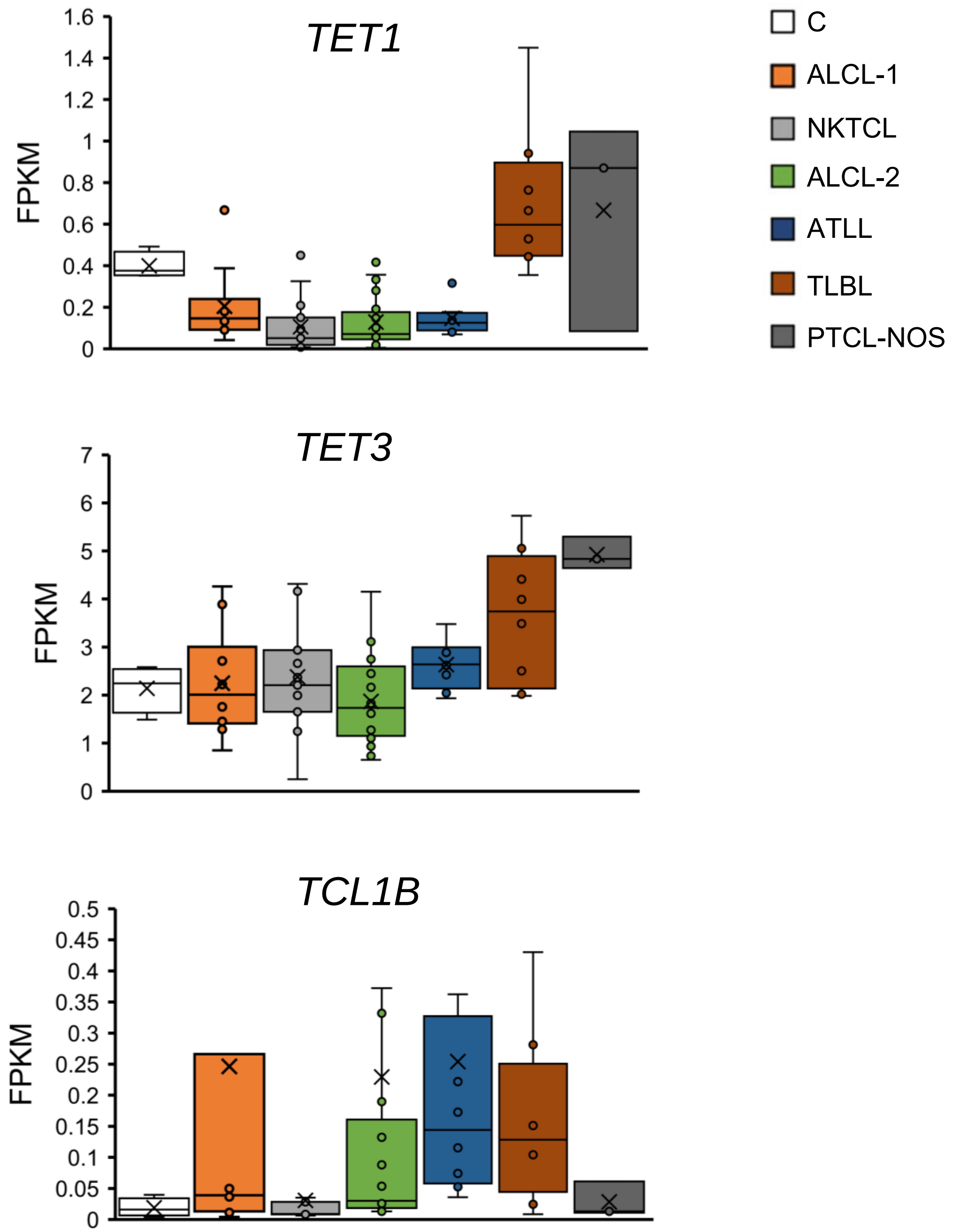

Figure S14. Comparative expression of *TET1*, *TET3*, and *TCL1B* in various T-cell lymphomas and control T-cells. *TET1, TET3* and *TCL1B* expression as determined by RNA-seq analysis of normal human T-cells (C, n = 4), two sets of human Anaplastic Large Cell lymphomas (ALCL-1, n = 5 tumors T1-T5 profiled in this study; ALCL-2, n = 21 publicly available data), NKTCL T-cell lymphoma lines (NKTCL, n = 15), adult T-cell leukemia/lymphomas (ATLL, n = 8), T-lymphoblastic lymphomas (TLBL, n = 8) and PTCL-NOS (n = 5, tumors T6-T10 profiled in this study). The horizontal line represents the median, bounds of the box-like range of variation, and whiskers- min and max values. *$p < 0.05$ (by DESeq2).

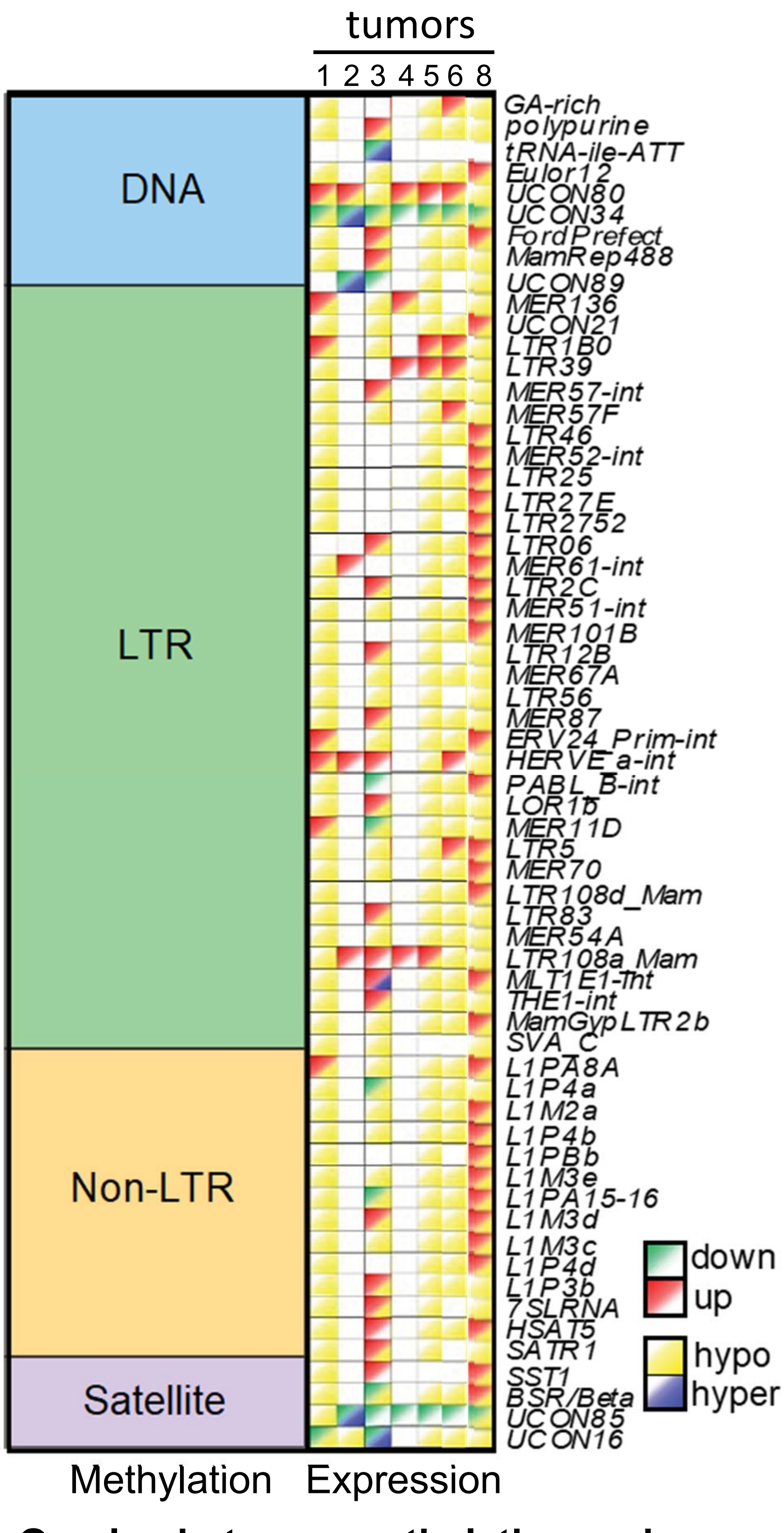

Figure S15. Overlap between methylation and expression of repetitive elements. Presented are all elements which methylation and expression was changed in at least one tumor. Expression of repetitive elements per family was calculated for each tumor relative to grouped CD4 and CD8 controls (n = 2 each) (FC > =2 and FDR < 0.05 by EdgeR).

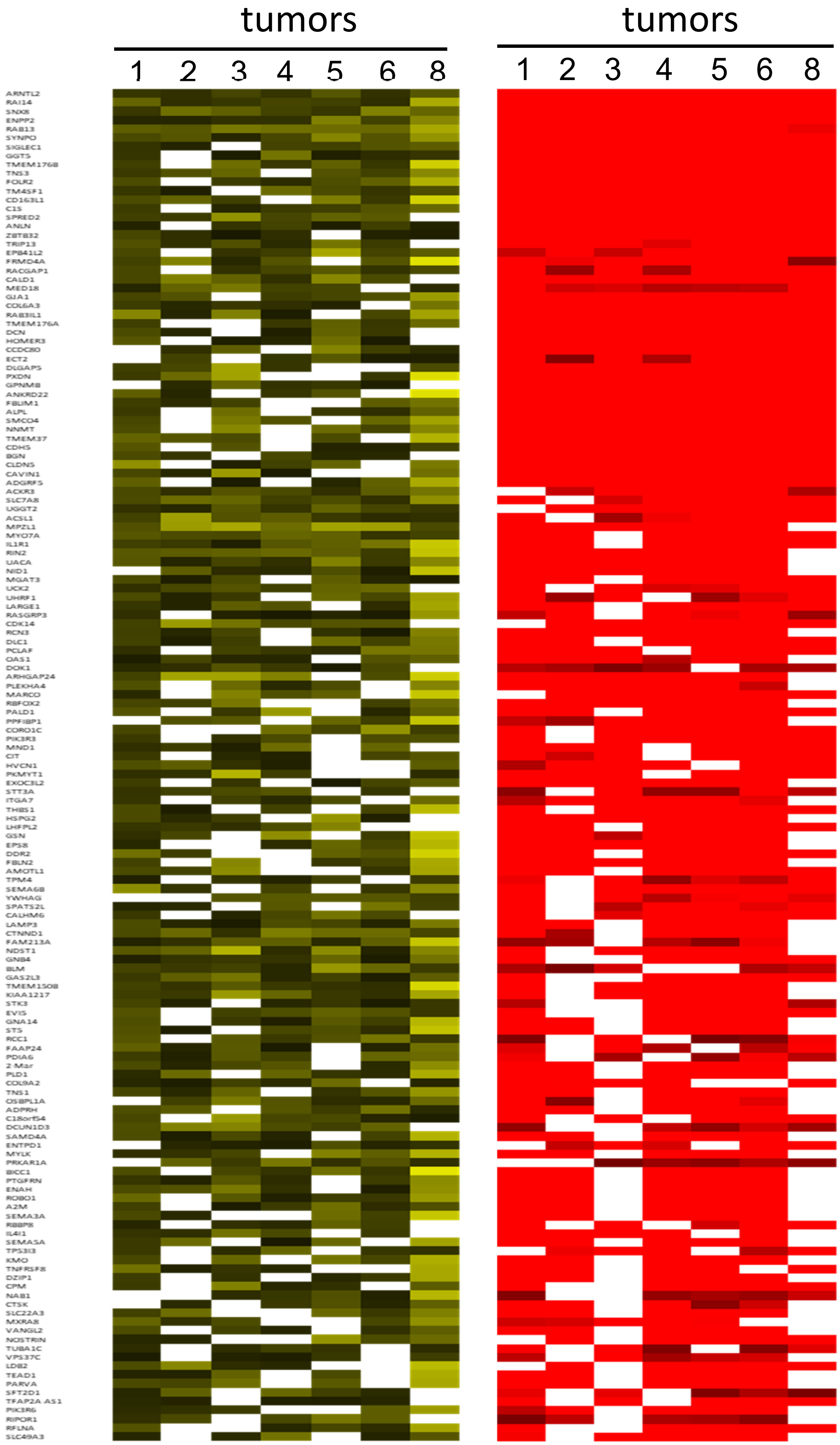

Figure S16. **Profile of hypomethylated and overexpressed genes in 5 out of 7 tumors.** There are 3,621 hypomethylated genes in 5 out of 7 tumors but only 2,810 have a matching gene in our expression analysis. Out of those 153 are also overexpressed in 5 out of 7 tumors (153/2,810 is 5.5%).

Term	Overlap	P-value	Odds Ratio	Combined Score	Genes
VEGFA-VEGFR2 Signaling Pathway WP3888	8/432	0.0183	2.5	10.12	CDH5;DOK1;TUBA1C;GJA1;CTNND1;ACKR3;LDB2;PDIA6
PI3K-Akt signaling pathway WP4172	7/340	0.0159	2.8	11.64	GNB4;PIK3R3;COL6A3;ITGA7;COL9A2;THBS1;PIK3R6
EGF/EGFR signaling pathway WP437	4/162	0.0360	3.3	11.12	EPS8;GJA1;PXDN;PLD1
Hippo-Merlin Signaling Dysregulation WP4541	5/120	0.0023	5.8	35.19	CDH5;PRKAR1A;ITGA7;TEAD1;STK3
Overview of leukocyte-intrinsic Hippo pathway functions WP4542	3/35	0.0024	12.4	74.70	RAB13;TEAD1;STK3
Pathways Regulating Hippo Signaling WP4540	4/98	0.0068	5.6	28.14	CDH5;PRKAR1A;TEAD1;STK3
Hippo-Yap signaling pathway WP4537	2/23	0.0132	12.5	54.08	TEAD1;STK3
Transcription co-factors SKI and SKIL protein partners WP4533	2/18	0.0082	16.4	78.84	TEAD1;STK3
Focal Adhesion WP306	5/198	0.0182	3.4	13.78	PIK3R3;ITGA7;PARVA;THBS1;MYLK
miRNA targets in ECM and membrane receptors WP2911	2/22	0.0121	13.1	57.91	COL6A3;THBS1
Microglia Pathogen Phagocytosis Pathway WP3937	2/40	0.0375	6.9	22.67	PIK3R3;PIK3R6
PKC-gamma calcium signaling pathway in ataxia WP4760	2/22	0.0121	13.1	57.91	GNA14;HOMER3
Complement system WP2806	3/97	0.0384	4.2	13.70	C1S;THBS1;DCN
Neural Crest Cell Migration in Cancer WP4565	2/43	0.0428	6.4	20.16	PIK3R3;PIK3R6
Regulation of Actin Cytoskeleton WP51	4/150	0.0283	3.6	12.92	ENAH;GSN;PIK3R3;MYLK
7q11.23 copy number variation syndrome WP4932	3/104	0.0457	3.9	12.07	CLDN5;VPS37C;FBLN2
Calcium Regulation in the Cardiac Cell WP536	4/150	0.0283	3.6	12.92	GJA1;PRKAR1A;GNB4;YWHAG
Myometrial relaxation and contraction pathways WP289	6/156	0.0013	5.4	35.67	GJA1;PRKAR1A;CALD1;GNB4;ACKR3;YWHAG
Nephrotic syndrome WP4758	3/45	0.0049	9.4	50.11	ANLN;SYNPO;ARHGAP24
Neural Crest Cell Migration during Development WP4564	2/40	0.0375	6.9	22.67	PIK3R3;PIK3R6
MED and Pseudoachondroplasia genes WP4789	1/6	0.0450	26.1	80.94	COL9A2

Figure S17. Pathway enrichment analysis of hypomethylated and overexpressed genes in tumors using EnrichR and WikiPathways. EnrichR and Wiki pathway enrichment analysis of 153 genes with increased expression and promoter hypomethylation in 5 out of 7 tumors shown in (**Fig. S16**).

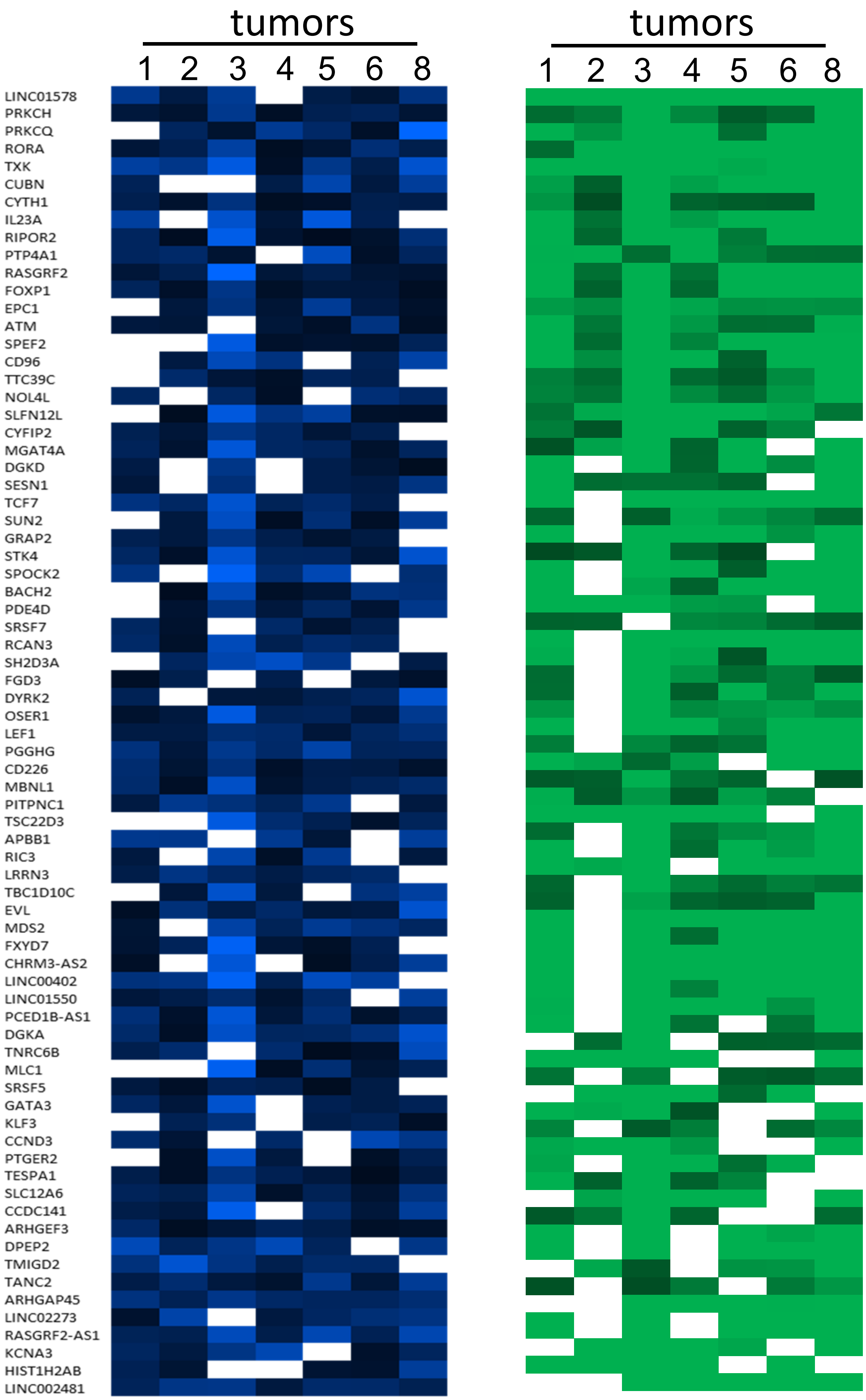

Figure S18. Profile of hypermethylated and downregulated genes in 5 out of 7 tumors. We identified 1,220 hypermethylated genes with the frequency of 5 out of 7 tumors out of which 74 are also downregulated in 5 out of 7 tumors**.** Decreased expression of these genes was associated with the deregulation of various pathways including the *Wnt signaling pathway and pluripotency* Pathways Regulating Hippo Signaling. (**Fig. S19).**

Term	Overlap	P-value	Odds Ratio	Combined Score	Genes
Wnt signaling pathway and pluripotency WP399	5/102	3.81E-05	14.81324	150.7329	CCND3;PRKCH;TCF7;LEF1;PRKCQ
Pathways Regulating Hippo Signaling WP4540	4/98	4.80E-04	12.05593	92.13889	PRKCH;TCF7;LEF1;PRKCQ
Wnt signaling WP428	3/115	0.008927	7.475101	35.27233	CCND3;TCF7;LEF1
Glioblastoma signaling pathways WP2261	3/82	0.003492	10.61526	60.05253	PRKCH;PRKCQ;ATM
Amplification and Expansion of Oncogenic Pathways as Metastatic Traits	2/17	0.001772	36.87222	233.613	LEF1;TCF7
Breast cancer pathway WP4262	3/154	0.01949	5.533532	21.79034	TCF7;LEF1;ATM
Endometrial cancer WP4155	2/63	0.022807	9.045993	34.2002	LEF1;TCF7
Chromosomal and microsatellite instability in colorectal cancer WP4216	2/73	0.029973	7.767997	27.24586	LEF1;TCF7
Modulators of TCR signaling and T cell activation WP5072	3/61	0.0015	14.47402	94.11267	DGKA;GRAP2;PRKCQ
T-cell receptor (TCR) signaling pathway WP69	3/90	0.004536	9.63526	51.98876	GRAP2;PRKCQ;GATA3
DNA damage response (only ATM dependent) WP710	4/110	7.41E-04	10.68464	77.00545	CCND3;TCF7;LEF1;ATM
G Protein Signaling Pathways WP35	3/93	0.004971	9.312676	49.39546	PRKCH;PDE4D;PRKCQ
G1 to S cell cycle control WP45	2/64	0.023487	8.899642	33.38537	CCND3;ATM
Thyroid stimulating hormone (TSH) signaling pathway WP2032	2/66	0.024872	8.62066	31.84486	CCND3;PDE4D
Ovarian infertility WP34	2/32	0.006235	18.42222	93.54167	PTGER2;ATM
Kisspeptin/kisspeptin receptor system in the ovary WP4871	2/39	0.00916	14.93168	70.07291	PRKCH;PRKCQ
Adipogenesis WP236	3/130	0.012437	6.587224	28.89869	MBNL1;RORA;GATA3
Endoderm differentiation WP2853	3/141	0.015447	6.058788	25.26722	SESN1;TCF7;LEF1
Mesodermal commitment pathway WP2857	3/147	0.017247	5.804577	23.56723	SESN1;LEF1;GATA3
Development and heterogeneity of the ILC family WP3893	3/32	2.23E-04	28.99029	243.7263	IL23A;RORA;GATA3
Myometrial relaxation and contraction pathways WP289	3/156	0.020159	5.460646	21.31902	PRKCH;PDE4D;PRKCQ
Non-genomic actions of 1,25 dihydroxyvitamin D3 WP4341	2/71	0.028476	7.993961	28.44799	PRKCH;PRKCQ
Arrhythmogenic Right Ventricular Cardiomyopathy WP2118	2/74	0.030733	7.659722	26.67428	LEF1;TCF7

Figure S19. Pathway enrichment analysis of downregulated and hypermethylated genes in tumors using EnrichR and WikiPathways. EnrichR and Wiki pathway enrichment analysis of 74 genes with decreased expression and promoter hypomethylation in 5 out of 7 tumors shown in (**Fig. S18**).

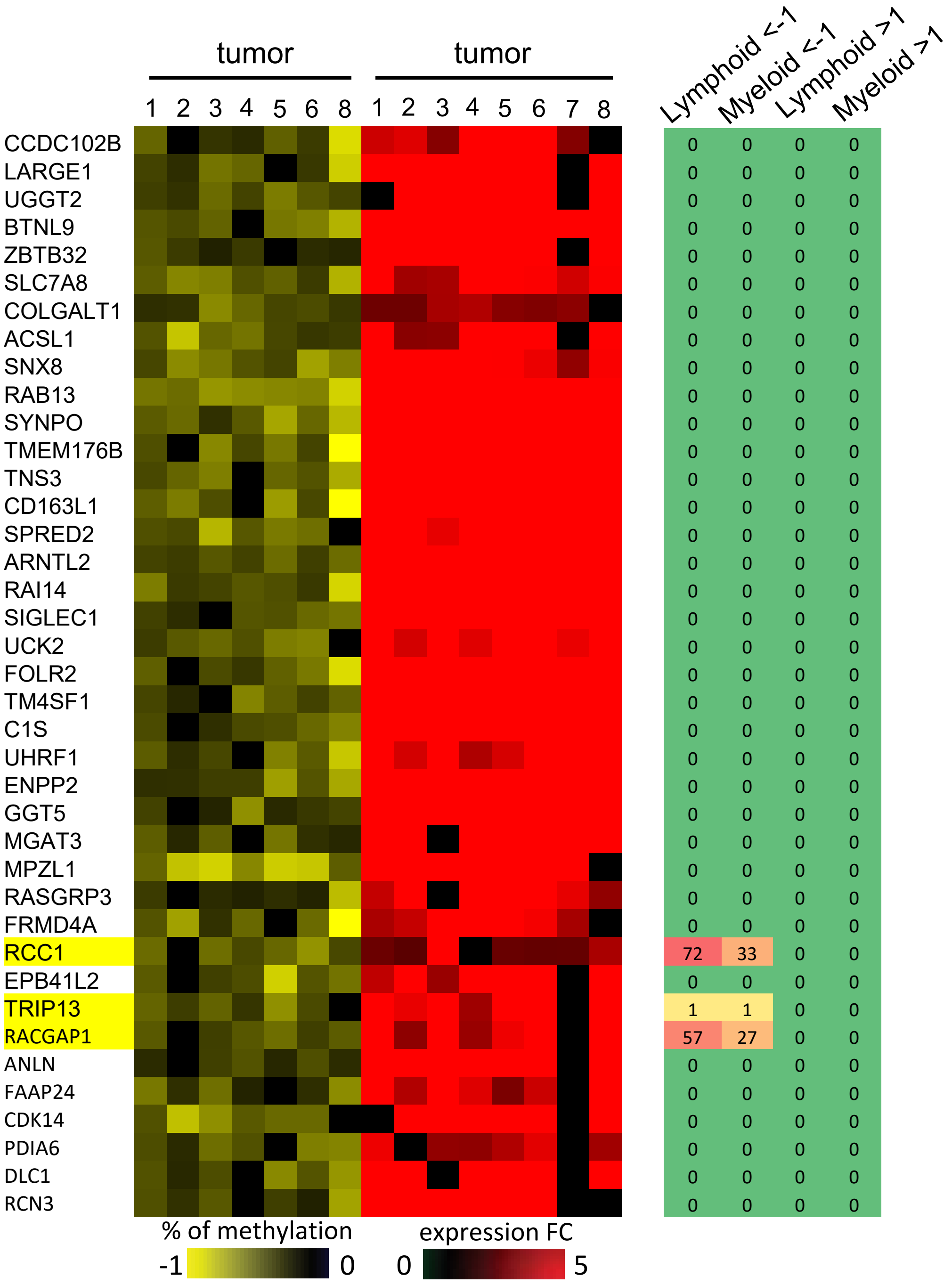

Figure S20. Impact of gene knockouts on hematologic cell line proliferation using CRISPR screens from the DepMap database. *RCC1, TRIP13* and *RACGAP1* are the only lethal genes in hematologic malignancies as determined by searching for phenotypes of genes using the DepMap database (Broad Institute, 39, 40) that contains results of CRISPR knockout screens in human cancer cell lines (Gene effects ≥ -1 were considered significant. The number of cell lines whose proliferation is negatively impacted by gene knockouts is shown.

Lane	T8ML-1	JURKAT	SUPT1	HH	MEC1	MEC2	RAJI
Target Protein	21440.69	19070.18	21678.004	9465.175	11604.52	7557.832	17173.25
Loading Control	20187.4	13766.93	12423.761	8354.054	11155.69	10730.64	13973.47
Ratio	1.062083	1.385216	1.7448826	1.133004	1.040233	0.704323	1.22899

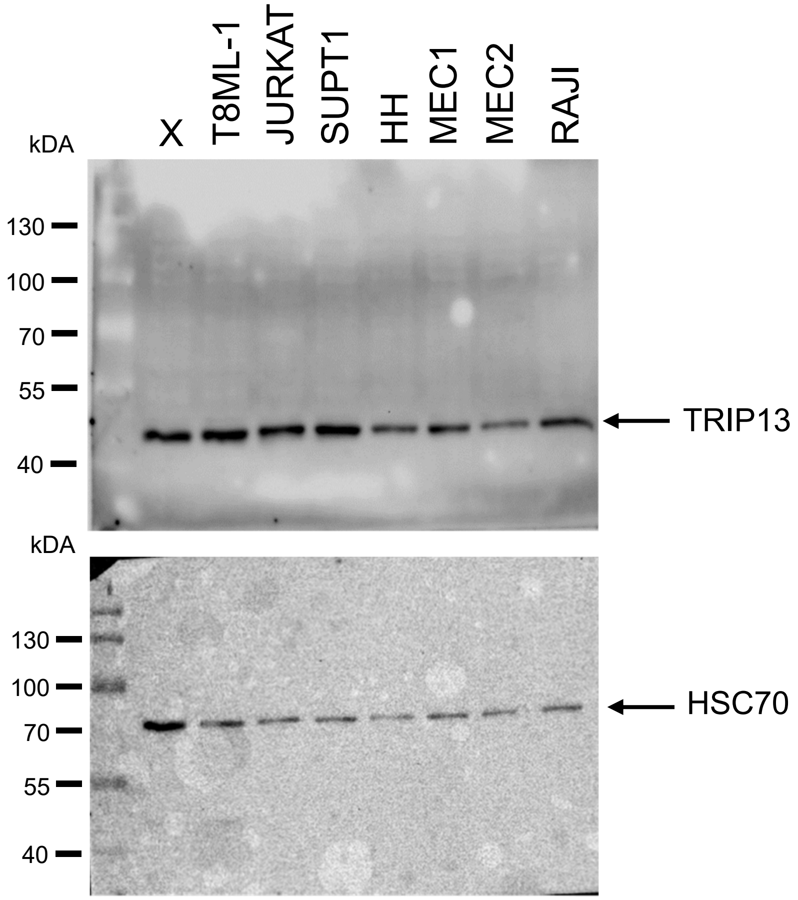

Figure S21. Uncropped version of western blot analysis of TRIP13 protein expression in hematologic cell lines shown in Figure 6C. Immunoblot analysis of TRIP13 protein levels in human lymphomas and leukemia cell lines. T8ML-1 (PTCL-NOS); JURKAT (Acute T-cell leukemia); SUP-T1 (T-cell lymphoblastic lymphoma); HH (Cutaneous T-cell leukemia/lymphoma), MEC1, MEC2 (Chronic B-cell leukemia); RAJI (Burkitt's lymphoma). HSC70 served as a loading control. Densitometric analysis ratios are shown for TRIP13 normalized to the loading control (HSC70). The table shows densitometric analysis of immunoblot signals.

Lane	K562	LOUCY	CCRF-CEM	MOT	MJ	DND41	MOLT4	CTV1
Target Protein	18530.15	16038.21	20508.276	19234.62	13695.45	20708.23	21268.74	21597.96
Loading Control	17096.45	16708.21	16408.619	18256.62	20211.21	16674.33	21853.15	19848.86
Ratio	1.08386	0.9599	1.2498478	1.05357	0.677617	1.241923	0.973257	1.088121

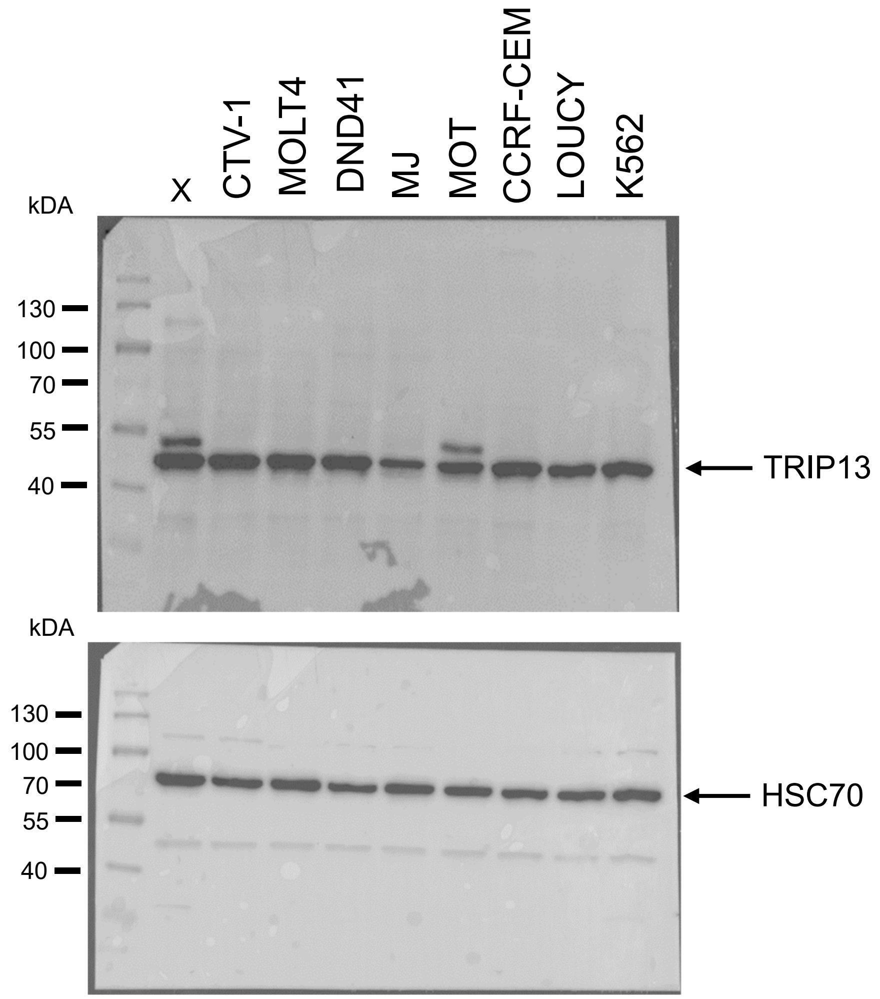

Figure S22. Uncropped version of western blot analysis of TRIP13 protein expression in hematologic cell lines shown in Figure 6C. Immunoblot analysis of TRIP13 protein levels in human lymphomas and leukemia cell lines. K-562 (Chronic myelogenous leukemia); LOUCY, CCRF-CEM (Acute T-cell lymphoblastic leukemia); Mo T (Hairy T- cell leukemia); MJ (Cutaneous T- cell lymphoma); DND41 (T-acute lymphoblastic leukemia); MOLT4 (T lymphoblast cell); CTV-1 (T-ALL). Densitometric analysis ratios are shown for TRIP13 normalized to the loading control (HSC70). The table shows densitometric analysis of immunoblot signals.

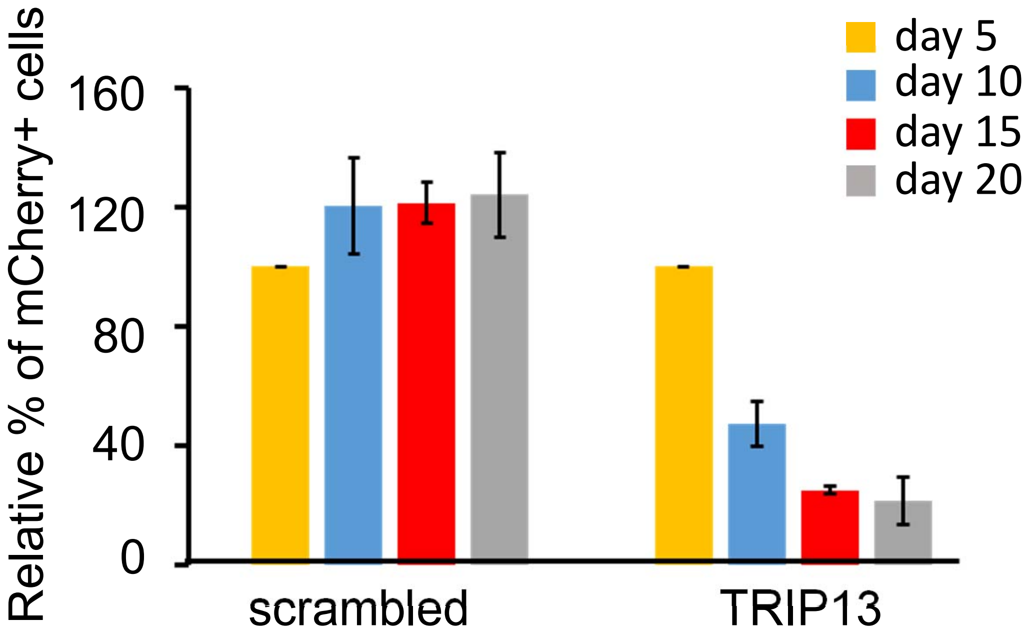

Figure S23. Time-course analysis of mCherry expression in T8ML-1 cells post-transduction with *TRIP13* shRNA-2 and scrambled control, as measured by FACS. Relative mCherry expression in T8ML-1 cells transduced with lentivirus encoding shRNA-2 against *TRIP13* or *scrambled*, measured by FACS 48 h after transduction (day 2) and thereafter upon continuous culturing *in vitro*. The transduction efficiency, as measured by mCherry expression at day 5, was set to 100% for both cells transduced with *scrambled* and *TRIP13* shRNAs. The values obtained for the percentage of mCherry-positive cells at each time point were plotted relative to the percentage at day 5. Data are presented as mean ±SEM (from two independent experiments) relative to *scrambled*, *p < 0.05. A two-tailed *Student's t-test* was used to calculate all p-values.

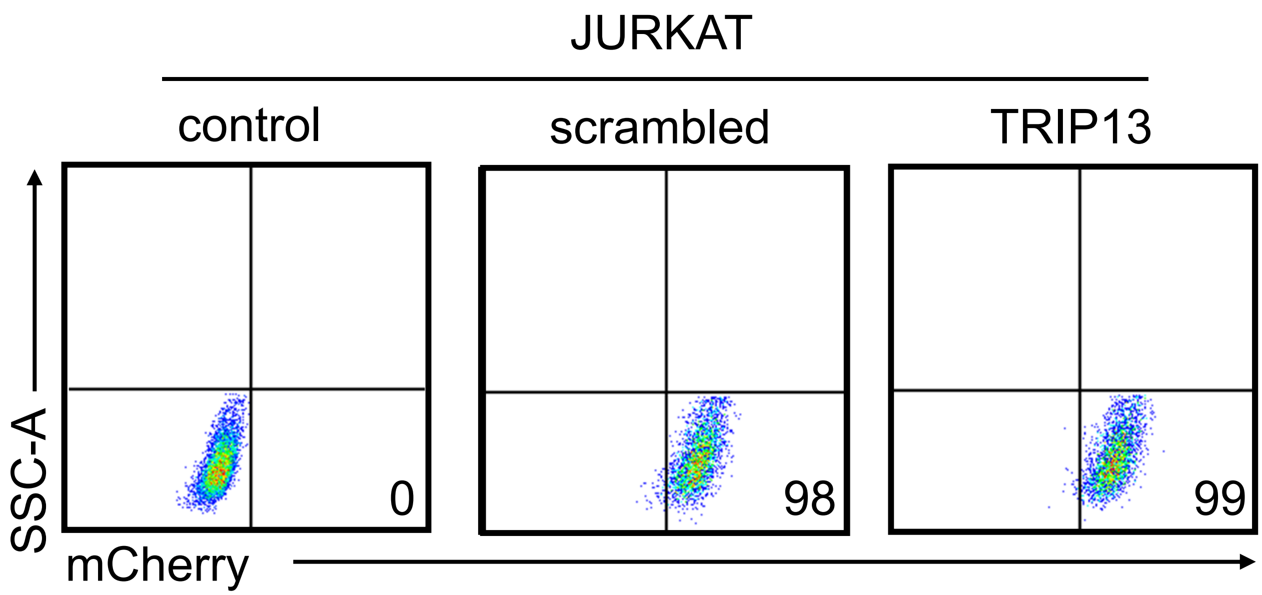

Figure S24. Assesment of transduction efficiency using Fluorescence-Activated Cell Sorting (FACS).
The leftmost panel, labeled “control", represents the negative control with JURKAT cells that have not undergone transduction, showing minimal to no fluorescence, indicating the baseline level of autofluorescence in the cell population. The central and rightmost panels, labeled “scrambled” and “TRIP13” respectively, show the percentage of transduced JURKAT cells that are expressing the marker at a level above the threshold set for positive selection. JURKAT cells can reach high levels of transduction efficiency, with up to 99% of the cells successfully transduced.

Lane	Scramble	TRIP13-1
Target Protein	28855.61	5010.841
Loading Control	33500.22	38843.28
Ratio	0.861356	0.129001
Ratios/control	1	0.149766

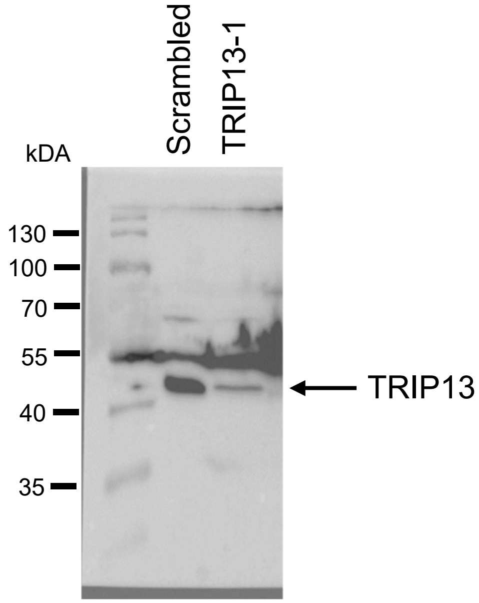

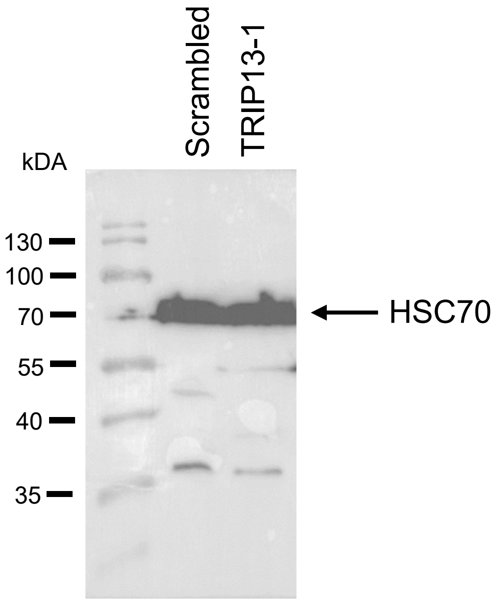

Figure S25. Uncropped version of western blot analysis of TRIP13 protein expression after shrna knockdown shown in Figure 7D. Immunoblot analysis of TRIP13 protein levels in JURKAT cells transduced with lentiviruses expressing either scrambled or TRIP13 shRNAs. Cells were harvested and analyzed 72 h after transduction. HSC70 served as a loading control. Densitometric analysis ratios are shown for TRIP13 normalized to the loading control (HSC70). The table shows densitometric analysis of immunoblot signals.

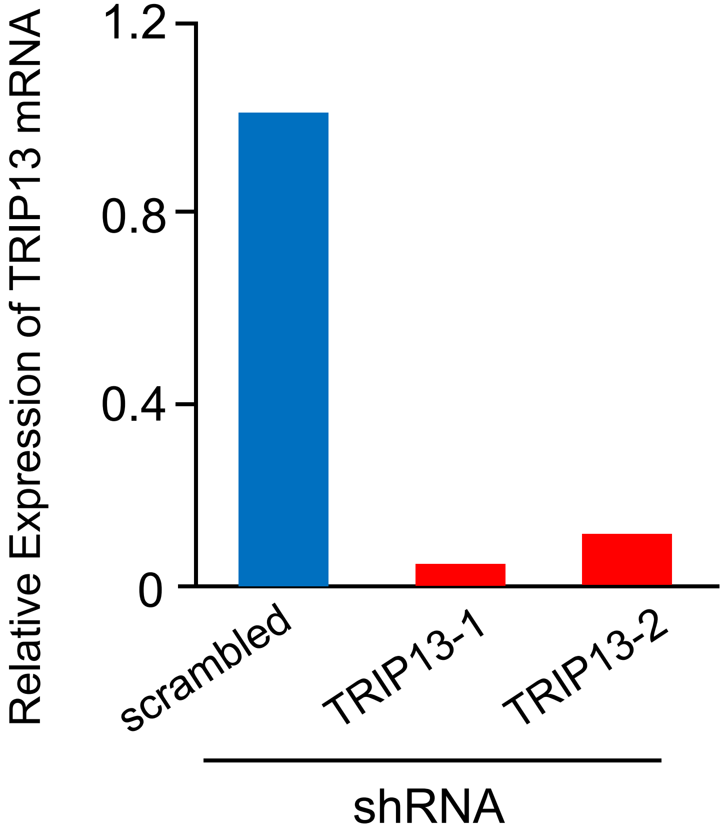

Figure S26. *TRIP13* shRNA-1 and shRNA-2 downregulate *TRIP 13* in JURKAT cells. JURKAT cells were transduced with lentiviruses encoding either scramble shRNA or shRNA-1 and shRNA-2 targeting *TRIP13*. At 72 h after transfection, total RNA was isolated from the cells and the relative expression level of target mRNA was determined by real-time RT-PCR analysis. Data are presented as mean values of two independent experiments with standard deviations. $p < 0.01$ compared with the control.

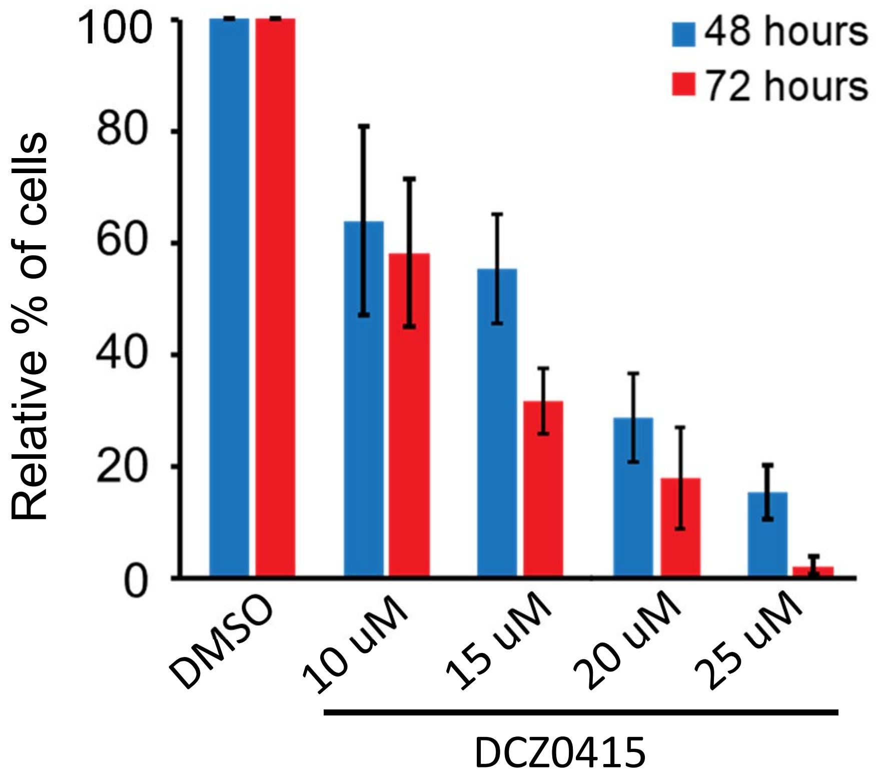

Figure S27. DCZ0415 treatment of T8ML-1 cells impairs proliferation *in vitro*. T8ML-1 cells were treated with either DMSO (vehicle) or DCZ0415 at indicated concentrations and counted using Trypan Blue exclusion dye. Bar graphs represent the total cell counts of live cells after 48 (blue) or 72 (red) hours of continuous drug treatment. Each data point is the average of three measurements taken in parallel and bar graphs represent ±SEM.

Lane	DMSO 48hr	DCZ0415 48hr	DMSO 72hr	DCZ0415 72hr
Cyclin B1	16122.217	24021.095	15521.338	29421.649
Loading Control	20108.004	21890.69	26952.347	26109.825
Ratio	0.80178107	1.097320139	0.57588076	1.12684206
Ratios/control	1	1.368603198	0.71825187	1.405423625

Lane	DMSO 48hr	DCZ0415 48hr	DMSO 72hr	DCZ0415 72hr
Cyclin D1	22792.288	30649.773	33731.773	33143.279
Loading Control	20108.004	21890.69	26952.347	26109.825
Ratio	1.13349331	1.400128228	1.25153379	1.269379592
Ratios/control	1	1.2352329	1.10413867	1.119882739

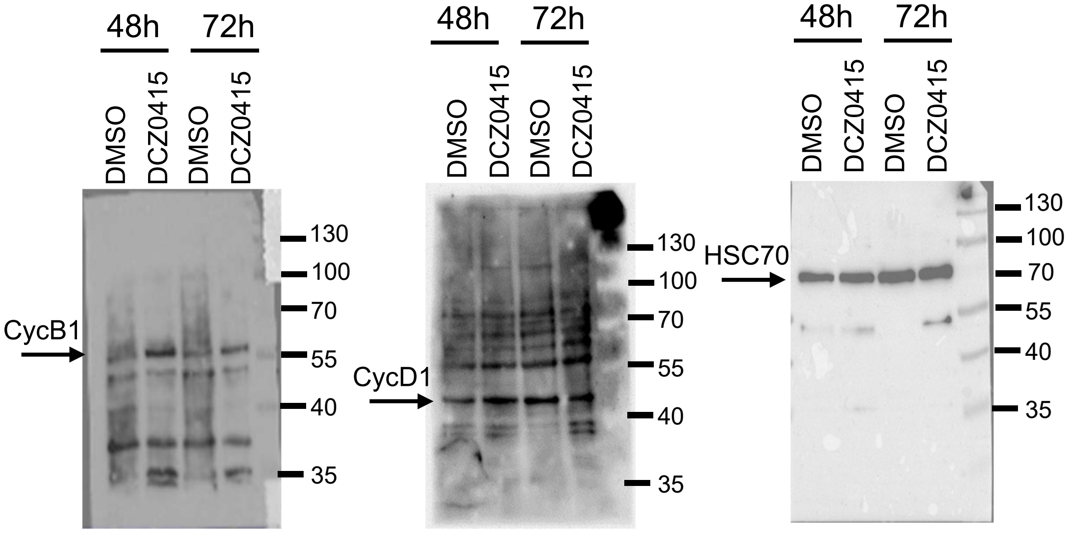

Figure S28. Uncropped version of western blot analysis of cell cycle proteins after DCZ0415 treatment shown in Figure 8E. Immunoblot analysis of Cyclin B1 and Cyclin D1 protein levels in T8ML-1 cell line treated with either DMSO (vehicle) or DCZ0415 harvested and analyzed after 48 hours and 72 hours of continuous drug treatment. HSC70 served as a loading control. Densitometric analysis ratios are shown for target proteins normalized to the loading control (HSC70). The table shows densitometric analysis of immunoblot signals.
